# From D-sorbitol to five-membered bis(cyclo-carbonate) as a platform molecule for the synthesis of different original biobased chemicals and polymers

**DOI:** 10.1038/s41598-018-27450-w

**Published:** 2018-06-14

**Authors:** Pierre Furtwengler, Luc Avérous

**Affiliations:** BioTeam/ICPEES-ECPM, UMR CNRS 7515, Université de Strasbourg, 25 rue Becquerel, 67087 Strasbourg, Cedex 2 France

## Abstract

Bis(cyclo-carbonate) was successfully synthesized from D-sorbitol (Sorb-BisCC) through an environmentally friendly process with dimethyl carbonate (DMC) as a reactant. In agreement with green chemistry principles, solvent free reactions were catalyzed and took place at low temperature. The reaction yield was increased until 50%, with the use of 1.3.5-triazabicyclo[4.4.0]dec-5-ene as catalyst and a continuous DMC feed to limit the side-reactions or the loss of reactant by azeotropic flux with a reactional subsidiary product. The obtained Sorb-BisCC is a remarkable platform molecule which could compete with others polycyclic platform molecules (isosorbide). Sorb-BisCC can be e.g., used to synthesize different chemicals such as short and long polyols, or novel biobased non-isocyanate polyurethanes (NIPU). Two Sorb-BisCC molecules have been coupled to obtain novel cyclic diols with pendant side chains. Polyether polyols were also obtained by anionic ring opening polymerization. According to the synthesis conditions, these synthetized polyether polyols range from partially to highly cross-linked materials. Finally, NIPU were synthesized with short and biobased fatty diamines. These different modifications and synthesis highlight the versatility of the Sorb-BisCC and demonstrated its high potential as building block. Sorb-BisCC can be considered as a platform molecule to open the way to different original and biobased chemical architectures.

## Introduction

D-sorbitol (Table 1) is a widely available and low-cost sugar alcohol which is mainly obtained from D-glucose hydrogenation^1^. D-sorbitol is a remarkable building block, selected by the Department of Energy (DoE-US) as one of the TOP 12 renewable value-added chemicals from biomass in 2004 or more recently in 2010^2,3^. It is for instance an important precursor for the synthesis of L-ascorbic acid (vitamin C)^4^. Sorbitol is widely used as food, drug and cosmetic additives and for the elaboration of chemical such as long polyols^5^.

**Table 1:** Chemical structures and properties of building blocks. D-sorbitol and different polycyclic sorbitol derivatives.

**Chemical structure**			
Chemical name	D-sorbitol	Isosorbide	Sorb-BisCC
Molar mass (g/mol)	182	146	216
Functionality	6	2	2
Functionality nature	Hydroxyl	Hydroxyl	Cyclic Carbonate
Melting temperature (°C)	95–100	61–62	214–216
C/O ratio	1	1.50	1.14
Annual production (Tons)	6.5 × 10^5^	Less than 10^3^	0

D-sorbitol has been directly used as monomer or building block in polyester synthesis through multistep pathways^6–8^. To develop eco-friendly synthesis process, D-sorbitol has also been involved in enzymatic reactions^9,10^. As previously reported, polyhydric chemicals were implicated in ester exchange reaction with a cyclic carbonate reactant such as diphenyl carbonate to obtained poly(cyclocarbonate)^11,12^. For example, D-mannitol tricarbonate was synthesized in severe conditions from D-mannitol and ethylene glycol at 150 °C under a reduced pressure of 20 mmHg^12^. Whereas, D-sorbitol tricarbonate was synthesized from diphenyl carbonate in N,N-dimethylformamide and catalyzed by sodium hydrogen carbonate at 110 °C^11^. More recently, such reactions were enhanced in agreement with the principles of a green chemistry^13^, with the use of another carbonate, dimethyl carbonate (DMC)^14^. DMC is an environmentally friendly reactive solvent according to its nontoxicity and represent an alternative to phosgene for methylation and carbonylation process^15^. Methoxycarbonylation with DMC is also an alternative route for carbamates and isocyanates synthesis^15^. Additionally, 50% of the total production of DMC is used for polycarbonate synthesis whereas 25% is used as solvent^16^. 85% of the DMC Europe’s production is based on the Enichem process^16–18^. The Enichem process uses carbon monoxide and oxygen as reactants, but its current production derived of CO_2_ is on the rise, positioning DMC as a green reactive solvent^16^. In this context, Tomczyk *et al*.^14^ used DMC instead of the diphenyl carbonate in reaction with D-sorbitol (10 eq.) catalyzed by potassium carbonate but 1,4-dioxane was used as solvent. The reaction product was a bis(cyclo-carbonate) (BisCC), the (1 R,4 S,5 R,6 R)-6-(1,3-dioxolan-2-one-4-yl)−2,4,7trioxa-3-oxy-bicyclo[3.3.0]octane, with a yield of 43% after recrystallization in acetonitrile instead of the D-sorbitol tricarbonate. Mazurek-Budzynska *et al*.^19^ also reported the synthesis of this molecule working with 10 eq. of DMC toward D-sorbitol in methanol catalyzed by potassium carbonate with a global yield of 40%. However, to the best of our knowledge, BisCC synthesis were always conducted in solvent conditions.

The D-sorbitol-based and five-membered bis(cyclo-carbonate) (Sorb-BisCC) is composed of two cyclo-carbonate groups and a tetrahydrofuran ring. It can be compared (Table 1) to another important polycyclic sorbitol derivative which is largely used as a building block with a large industrial production: the isosorbide^20,21^. Isosorbide results from a double dehydration of the sorbitol, which directly influences the carbon/oxygen ratio (C/O) (Table 1). The C/O ratio of Sorb-BisCC (Table 1) indicates a conservation of the richness in oxygen atoms from D-sorbitol (close to 1) compared to isosorbide, for instance. Obviously, the annual production and price of the Sorb-BisCC cannot be assessed as it is not produced at industrial scale.

Isosorbide has been largely developed and investigated as an innovative biobased rigid diol during the two last decades^22^. For instance, polyesters such as polyisosorbide terephthalate (PIT) or polyethylene isosorbide terephthalate (PEIT) were developed^20^. The replacement of ethylene glycol by isosorbide improved the rigidity and the behavior of the final polyester. Tg of PIT is 200 °C whereas the Tg of PEIT evolved from 70 to 180 °C^21,23^.

Sorb-BisCC presents a similar molar mass than isosorbide with also a bifunctionality. However, its melting temperature (T_m_) is more than three time the isosorbide’ one. This higher T_m_ indicates a strong rigidity and thermal resistance of the Sorb-BisCC. As isosorbide which can be directly valorized towards biobased polymers by esterification^24^ or Williamson etherification^25^, Sorb-BisCC molecule can open a variety of routes with diverse polymerization types. Sorb-BisCC is a versatile molecule which can be considered as a platform molecule to extend the development of advanced oligomers and polymers such as non-isocyanate polyurethanes (NIPU)^26–29^, polyol polyethers^30^, or by ring opening polymerization (ROP) to obtain various macromolecular architectures such as polycarbonates^31^.

BisCC can be for instance used for the synthesis of aliphatic polycarbonates at low temperature (≤60 °C) via anionic ROP^32,33^. These authors assumed that the reactivity is linked to the particular ring strain to polymerize at low temperature. Such low temperatures have been only reported on aldohexopyranosides monomers^33^. Most of the ROP based on BisCC are thermodynamically unfavorable and operate at high temperature (≈150 °C) leading to the elimination of carbon dioxide to produce linear copolymers with carbonates and ethers groups.

On the same way, the synthesis of non-isocyanate polyurethanes (NIPU) can rely on BisCC. NIPU are mainly synthesizing according to three types of reaction which are (i) AB-type azide condensation^34^, (ii) transurethane polycondensation^35^ and (iii) aminolysis^36^. The aminolysis reaction is based on the reaction between a cyclocarbonate and an amine function. It is until now, the most promising way to avoid the use of toxic polyisocyanate in polyurethane synthesis. Resulting materials are called polyhydroxyurethanes (PHU), as hydroxyl groups are generated, close to the urethane group^37^. BisCC presents a lower reactional rate toward amine functions than six-membered cyclic carbonate but they are widely used^38^. Compared to six, seven or even eight-membered cyclic carbonate, BisCC are more economically viable as they are easily obtained with high yield and high renewable content^39–41^. Additionally, their synthesis does not require phosgene derivatives whereas six-membered cyclic carbonate does^42^.

The aim of this study was to synthetized a biobased platform molecule, BisCC from an attractive renewable building-block, the D-sorbitol. The Sorb-BisCC synthesis parameters were investigated and enhanced to improved D-sorbitol conversion and limit side reactions with respect to green chemistry principles (solvent-free, low temperature and catalyzed reactions). To show that Sorb-BisCC is a platform molecule, it was used as a basis leading to the elaboration of different products. Synthetized Sorb-BisCC was implicated in the elaboration of different and new biobased molecular architectures such as a new diol, cross-linked polyethers and NIPU with also renewable diamines. All products from the Sorb-BisCC to its derivatives chemical structures and properties were fully characterized and analyzed.

## Materials and Methods

D-sorbitol was kindly provided by Tereos (Meritol, 98%, water content <0.5%, reducing sugar content <0.1%). 1.3.5-triazabicyclo[4.4.0]dec-5-ene (TBD), 1.8-diazabicyclo[5.4.0]undec7-ene (DBU), tert-butylimino-tri(pyrrolidino)phosphorarane (purity ≥97%, BTPP), dimethyl carbonate (purity ≥99%, DMC), diethyl carbonate (purity ≥99%, DEC) ethylene carbonate (purity 99 + %, EC), 1-octanol (purity ≥ 99%), cis-1,2-cyclopentanediol (98%), (±)-trans-1,2-cyclopentanediol (97%) and 2-chloro-4,4,5,5-tetramethyl-1,3,2-dioxaphospholane (Cl-TDP, 95%) were obtained from Sigma Aldrich. Sodium hydroxide (NaOH) was obtained from Carlo Erba Reagents. Potassium Hydroxide (KOH), potassium carbonate (K_2_CO_3_) and 1.5-diamino-2-methylpentane (1.5 MD, 99%) were obtained from VWR Chemical. Titanium (IV) isopropoxide (TTIP), and titanium (IV) butoxide (TNBT) were obtained from Acros Organics. 1,4 diaminobutane (1.4 B, 98 + %) and stannous octoate (Sn(oct), 95%) were obtained from Alfa Aesar. Hexamethylene diamine (1.6 H, 98%) was provided by BASF. A diamine based on dimer fatty (DDA), obtained from the dimerization of two fatty acids^43^ was kindly provided by Croda (Priamine 1075). At room temperature, DDA is a yellowish, slightly viscous liquid. Some DDA properties are summarized in Table 2. All reagents were used without further purification.

**Table 2:** DDA main properties^63^.

Designation	Amine Value (mmol/g)	Functionality	Dimer content (wt%)	Carbon chain length	Tg (°C)
DDA	3.64	2.0	˃99	36	<−50

### Synthesis with catalyst of D-sorbitol-based Bis(cyclo-carbonate)

A round-bottom flask equipped with a distillation bridge or vigreux fractionating columns, or reflux device was charged with one molar equivalent of D-sorbitol. 5 mol% of catalyst (TBD, DBU, BTPP, KOH, NaOH, K_2_CO_3_, TTIP, TNBT or stannous octoate (Sn(oct)) were added, in respect to D-sorbitol (Table 3).

**Table 3:** Sorb-BisCC synthesis from D-sorbitol and DMC or EC, under different conditions and catalyst systems.

Entry	Reactional device	D-sorbitol/carbonate molar ratio	Catalyst	Reaction time (h)	Yield (%) Synthesis of Sorb-BisCC
1	Distill. Bridge	4^a^	None	14.5	18
2	Distill. Bridge	3.5^b^	TTIP	16	0
3		3.5^b^	TNBT	16	0
4		3.5^b^	Sn(oct)	16	0
5		3.5^b^	KOH	16	0
6		3.5^b^	NaOH	16	0
7		3.5^b^	K_2_CO_3_	16	0
8		3.5^b^	BTPP	1–16	21
9		3.5^b^	DBU	1–16	24
10		3.5^b^	TBD	1–16	22
11		7	BTPP	16	35
12		9	BTPP	1–16	42
13		5^b^	TBD	1–16	40
14		7^b^	TBD	1–16	41
15	Reflux	3.5^b^	TBD	16	10
16	Vigreux.	3.5^b^	TBD	16	15
17		5	TBD	16	14
18		7^b^	TBD	1–24	36
19		9^b^	TBD	1–48	45
20		12^b^	TBD	16	Mainly byproducts
21		15^b^	TBD	16	Mainly byproducts
22		18^b^	TBD	16	Mainly byproducts

^a^EC, under vacuum reaction.

^b^DMC.

Then, different amounts of DMC, comprised between 3.5 and 18.0 molars equivalents with respect to D-sorbitol, were added. In the case of continuously feed reaction, a syringe pump (Fischer Scientific No 9034914) was used to keep constant the DMC flow rate (Table 4). The mixture was stirred and heated to 75 °C, for 16 to 48 h (Tables 3 and 4). At the end, the media was cool down to room temperature. Then, distilled water was added to precipitate the product. Reaction products were filtrated over 0.45 μm polyvinylidene fluoride (PVDF) membrane and wash with distilled water. Finally, the product was dry overnight at 50 °C under vacuum. When reaction kinetic was investigated, each yield was calculated from a dedicated reaction.

**Table 4:** Synthesis of Sorb-BisCC from D-sorbitol using a continuous feed of DMC.

Entry	DMC initial feed in respect to D-sorbitol (mole/mole)	Aditional feed of DMC, in respect to D-sorbitol (mole/mole)		Reaction total time (h)	Yield (%)
		Molar ratio	Flow rate (mL/h)		
1	3.35	3.35	0.15	16	50
2	3.35	6.70	0.30	16	43
3	1.00	6.70	0.30	16	35

### Synthesis without catalyst of D-sorbitol-based Bis(cyclo-carbonate)

A 50 mL round-bottom flask equipped with a distillation bridge was charged with one molar equivalent of D-sorbitol and four molar equivalents of EC. The mixture was stirred, heated to 150 °C, and maintained under controlled vacuum (23–33 mbar) to remove reaction byproducts (ethylene glycol). Different reaction times were investigated (6, 14.5, 24 or 48 h). A brown mixture was obtained at the end. The medium was cool down to room temperature and brought back to atmospheric pressure. 10 mL of ethanol were added and the medium was heated until full dilution in ethanol. The mixture was then poured into a beaker and let for two days at 4 °C, for crystallization. Brown crystals were obtained and recovered by filtration. They were finally washed with glacial acetic acid to obtain white crystal and then dried overnight, at 50 °C under vacuum.

### Synthesis of cyclopentanediol by trans-esterification

A 25 mL round flask equipped with a distillation bridge was charged with one molar equivalent of cis-1,2-cyclopentanediol or trans-1,2-cyclopentanediol, 3.5 molar equivalent of DMC with respect to the diol and 5 mol% equivalent to the diol of TBD. The mixture was stirred and heated at 75 °C for 16 h. At the end, the DMC excess was distilled under vacuum. The corresponding product and the residual TBD were directly analyzed, without further purification.

### Synthesis of polyether polyols

A 50 mL round flask was charged with different molar ratio of Sorb-BisCC and 1-octanol at 150 °C, for 2 to 24 h with 0.7 or 5 mol% of K_2_CO_3_ as a catalyst, regarding Sorb-BisCC content (Table 5). 1 mL of dimethylsufoxide was added as solvent. Then, the flask was closed with a septum. The reactional system was flush under argon for 10 minutes before launching. During the reaction, the septum was pierced with a needle to evacuate the potential gaseous coproducts. The final product was a dark product. In most cases, the product was a liquid, which was precipitated in 50 mL of toluene. The solid materials were washed in 50 mL of water.

**Table 5:** Conditions of synthesis to obtain polyether/polycarbonate from Sorb-BisCC.

Entry	Initiator	Initiator/Sorb-BisCC molar ratio	K_2_CO_3-_ catalyst (%mol)	Temperature (°C)	Time (h)
1	1-octanol	1/1	5%	150	2
2	1-octanol	1/100	5%	150	17
3	1-octanol	1/50	5%	150	24
4	1-octanol	1/25	5%	150	17
5	1-octanol	1/100	0.7%	150	2
6	1-octanol	1/100	0.7%	150	24
7	1-octanol	1/50	0.7%	150	24
8	1-octanol	1/25	0.7%	150	24
9	1-octanol	1/10	0.7%	150	24

### NIPU synthesis

A 50 mL round flask was charged with different diamines such as 1,4 diminobutane, 1,2-diamino-2-methylpentane, 1,6 hexamethylene diamine, DDA with an equimolar ratio of Sorb-BisCC (Table 6). 10 mL of methanol were added as a solvent^44^. Then the medium was heated until reflux temperature (66 °C) for 72 h. Then, the methanol was eliminated by evaporation. The viscous product was pour on a non-adhesive Teflon® sheet and placed into an oven at 80 °C for 72 additional hours before being hot pressed at 80 °C, to obtain material plates. Then, two series of NIPU with a blend of DDA with 1.4 B or 1.5 MD were prepared according to the same protocol (Table 6).

**Table 6:** PHU names, compositions and properties.

Entry	Sample name	Diamine 1	Diamine 2	Diamine 1/Diamine 2 (Molar ratio)	T_deg 50%_ (°C)	Tg (°C)	OH-Value (mmol /g)	Mn (g/mol)	Mw (g/mol)	Đ
1	PHU-P	DDA	none	1/0	359	−3	3.25	2430	5370	2.2
2	PHU-B	1.4 B	none	1/0	216	41	8.84	1910	4480	2.4
3	PHU-D	1.5 MD	none	1/0	217	42	8.69	2270	5850	2.6
4	PHU-H	1.6 H	none	1/0	224	41	4.83	1450	2720	1.9
5	PHU-0.2B	DDA	1.4 B	0.8/0.2	318	−9	3.90	1820	3120	1.7
6	PHU-0.4B	DDA	1.4 B	0.6/0.4	326	−4	4.58	1930	4360	2.3
7	PHU-0.6B	DDA	1.4 B	0.4/0.6	280	12	5.26	1840	4190	2.3
8	PHU-0.8B	DDA	1.4 B	0.2/0.8	257	20	6.26	1690	5030	3.0
9	PHU-0.2D	DDA	1.5 MD	0.8/0.2	304	−2	4.06	2770	5040	1.8
10	PHU-0.4D	DDA	1.5 MD	0.6/0.4	324	2	4.48	2140	4870	2.3
11	PHU-0.6D	DDA	1.5 MD	0.4/0.6	276	20	5.15	2400	5520	2.3
12	PHU-0.8D	DDA	1.5 MD	0.2/0.8	270	37	6.01	1500	5180	3.5

### Acetylation of samples

To enhance samples solubility in tetrahydrofuran for SEC analysis, acetylation of the hydroxyl groups was performed when required. Acetylation was done in a pyridine/acetic anhydride mixture (1:1 v/v) at room temperature for 24 h to increase sample solubility for analysis as previously reported^45^.

### Characterizations

^1^H- and ^13^C-NMR spectra were performed with a Bruker 400 MHz (US). Deuterated dimethyl sulfoxide (DMSO-*d*_6_) was used as solvent to prepare sample solutions with concentrations of 8–10 and 20–30 mg/mL for ^1^H-NMR and ^13^C-NMR, respectively. The number of scans was set to 64 for ^1^H-NMR and at least 2048 for ^13^C-NMR. The calibrations of ^1^H- and ^13^C-NMR spectra were performed using the DMSO peak at 2.50 and 39.52 ppm, respectively. The water molecules present in DMSO-d_6_ eventually introduce an additional peak in ^1^H-NMR spectra at 3.33 ppm.

^31^P-NMR analyses were performed with a Bruker 400 MHz (US) spectrophotometer after phosphitylation of the sample with Cl-TDP according to standard protocols^46,47^, the number of scans was set to 128 at 25 °C. Peak analysis and quantitative analysis were performed according to previous reports^48^.

Elementary analyses were performed on a ThermoFisher Scientific “Flash 2000” (US) device (absolute precision of 0.3%) with 1 mg sample burned up to 950 °C.

Electrospray ionization mass (MS) experiments were performed on a Bruker Daltonics microTOF spectrometer (Bruker Daltonik GmgH, Bremen, Germany) equipped with an orthogonal electrospray interface (ESI). Calibration was performed using a solution of 10 mM sodium formiate. Sample solutions were introduced into the spectrometer source with a syringe pump (Harvard type 55 1111: Harvard Apparatus Inc., South Natick, MA, USA) with a flow rate of 5 µL.min^−1^.

Differential scanning calorimetry (DSC) was performed on a TA Instrument Q200 (Q5000 (TA Instruments, US) under nitrogen flux (50 mL/min). Samples of 1–3 mg were sealed in hermetic aluminum pans and analyzed using cyclic procedure involving a heating ramp comprises between −50 to 260 °C at 10 °C/min, a cooling ramp at 5 °C/min, then a second heating up to 260 °C at 10 °C/min. Between each ramp, the temperature was held 2 min for stabilization. For the DSC analysis of PHU, the heating ramp does not exceed 100 °C.

Thermogravimetric analyzes (TGA) were performed using a TA Instrument Hi-Res TGA Q5000 (TA Instruments, US) under reconstituted air (flow rate 25 mL/min). Samples of 1–3 mg were heated from room temperature to 700 °C (10 °C/min). Characteristic temperature of 5%wt (T_deg5%_) and 50 wt% (T_deg50%_) of weight of loss were noted. The main characteristic degradation temperatures were those at the maximum of the weight loss derivative curve (DTG) (T_deg_).

Infrared spectroscopy was performed with a Fourier transformed infrared spectrometer Nicolet^TM^ 380 from Thermo Scientific (US) used in reflection mode equipped with an ATR diamond module (FTIR). An atmospheric background was collected before each sample analysis (32 scans, resolution 4 cm^−1^).

Size exclusion chromatography (SEC) measurements were performed in tetrahydrofuran (THF, HPLC grade) in Waters (US) Acquity APC system equipped with a 1.7 µm, 45 Å 150 mm APC XT column, 2.5 µm, 200 Å 150 mm APC XT column and a 2.5 µm, 450 Å, 150 mm APC XT column, a Acquity RI refractive index detector and a Acquity TUV diode array (UV) detector. The instrument was calibrated with linear polystyrene standards from 162 to 1,650,000 g/mol and reported molar masses are the molar masses at the peak. Sample presenting low solubility in THF were acetylated prior to the performed analysis. In the case of small molar masses (i.e. oligomers) the degree of polymerization (DPn) is evaluated according to equation (1).

1 $$DPn=\frac{(Mn-M{n}_{ini})}{M{n}_{0}}$$

Where Mn is the obtain number-average, Mn_ini_ the molar masses of the initiator and Mn_0_ the molar masses of the monomer.

## Results and Discussion

### Synthesis of Sorb-BisCC

**Figure 1:**
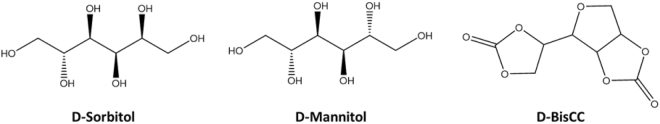
D-sorbitol, D-mannitol and Sorb-BisCC structures.

In order to study the D-sorbtiol conversion into Sorb-BisCC several reactional conditions and setting were investigated and detailed in Table 3. The product of each successful reaction presented in Table 3, entry 8 to 19 was a white powder directly obtained by precipitation in water, with the exception of the product presented in Table 3, entry 1 which was recovered after re-crystallization (white powder too). Elementary analysis and ESI results were in perfect agreement with the values obtained from the theoretical Sorb-BisCC chemical structure (Fig. 1), as presented in Table 7.

**Table 7:** ESI and elementary analysis of Sorb-BisCC. Theoretical and experimental results.

		Theoretical values	Results
Elementary Analysis	Carbon (%)	44.46	44.61
	Hydrogen (%)	3.73	3.88
	Nitrogen (%)	0.00	0.00
ESI	[M + K^+^] (g/mol)	255.00	254.99

The absence of nitrogen in the product is a strong argument which shows the efficiency of the removal of TBD and DBU. The DSC analysis of Sorb-BisCC is given on the Supplementary information (SI) with Fig. S1. This Figure shows a sole endothermic peak at 214 °C corresponding to the fusion. FTIR spectra (Supplementary Fig. S2) present typical C=O stretching vibration of five membered cyclic carbonate at 1778 cm^−1^ and the peak at 1130 cm^−1^ is related to the C-O-C stretch. Finally, C-H_2_ and C-H linkage stretching vibration are visible at 2995–2992 and 2880 cm^−1^, respectively. The Sorb-BisCC structure was confirmed by ^1^H, ^13^C and HSQC-NMR, (Supplementary Fig. S3a–c, with fully attributed signals).

^1^H-NMR, δ (ppm): 5.4 (m, 2 H, CH-CH(O)), 5.0 (m,1 H, CHO), 4.6 (t, 1 H, CH_2_O), 4.5 (m, H, CH_2_O), 4.2 (d, 1 H, CH_2_O), 4.0 (d, 1 H, CHO), 3.8 (d, 1 H, CH_2_O), (fully attributed ^1^H-NMR spectra available in Supplementary Fig. S3a).

^13^C-NMR, δ (ppm): 154.7 (C=O), 153.9 (C=O), 81.1 (CH-O), 80.8 (CH-CHOC(O)), 79.6 (CH2-CHO-C(O)), 74 (CHO-C(O)-CH2O), 71.8 (CH2O), 66.3 (CH2O) (fully attributed ^13^C-NMR spectra available in Supplementary Fig. S3b).

According to all these different characterizations, the unique synthesized product was the Sorb-BisCC.

A preliminary protocol has been tested with the same conditions previously described on the synthesis of tri-cyclocarbonate from D-mannitol^12^, but in this case with the D-sorbitol as a starting reagent (Table 3, Entry 1). The corresponding conditions (high temperature, controlled vacuum) are not adequate and did not permit the obtaining of tri-cyclocarbonate. After recrystallization, the obtained product was Sorb-BisCC with a low yield (18%), largely lower than the one previously reported with D-mannitol (65%). Contrary to the D-mannitol, D-sorbitol has a high tendency to undergo a first dehydration with these conditions. This is explained by the stereochemistry differences of both isomers (Fig. 1). In the D-sorbitol, several hydroxyl groups present *cis* configuration along the backbone. In the case of D-mannitol, three pairs of hydroxyl groups present three distinct planes. This last configuration highly promotes the formation of five-membered cyclocarbonate.

**Figure 2:**
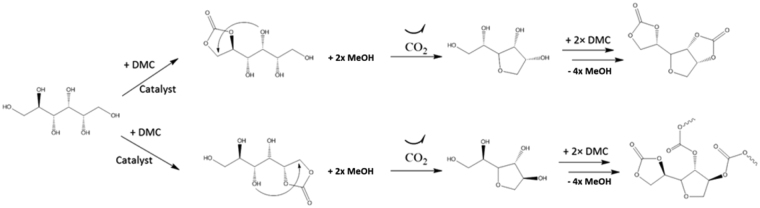
Proposed reaction path-way of D-sorbitol with DMC, from Mazurek-Budzynsky *et al*.^19^.

Mazurek-Budzynsky and al.^19^ also faced a limited D-sorbitol conversion into Sorb-BisCC when reacted with DMC. These authors proposed a reactional pathway (Fig. 2) with the dehydration of the D-sorbitol firstly occurs and with two reactional intermediates, 1,4- and 3,6-sorbitan, respectively. However, only the pairs of diols of 1,4-sorbitan, in *cis* configuration, can undergo the transesterification with DMC. Whereas, diols in *trans* configuration are not suitable for the formation of five-membered cyclic carbonates because of the ring strength which lead to linear carbonates. Our observations are on perfect agreement with Mazurek-Budzynsky and al.^19^ results, leading to the same conclusions. The stereochemistry and the number of available diols strongly affect the formation of five-membered cyclocarbonates, regardless the structure (linear or cyclic) of the carbonates (DMC or EC, respectively) involved in the transesterification.

To confirm the previous hypothesis concerning the effect of the spatial configuration of the hydroxyl groups on the final yield, different model molecules based on cis-1,2-cyclopentanediol (^1^H-NMR spectrum available in Supplementary Fig. S4) and trans-1,2-cyclopentanediol (^1^H-NMR spectrum available in Supplementary Fig. S5) corresponding to the cyclic diols present in 3,6-sorbitan and 1,4-sorbitan, respectively, were studied. ^1^H-NMR signal attribution (Supplementary Fig. S6) of the synthesis product obtained from cis-1,2-cyclopentanediol was a cyclic carbonate, with a typical chemical displacement of δ (ppm) = 5.4 (m, 2 H, CH-CH(O)). Additionally, the absence of the alcohol signal at δ (ppm) = 4.2 (m, 2 H, CH(OH)) is a strong confirmation of the full conversion of the cis-1,2-cyclopentanediol. Instead, ^1^H-NMR signals attributions (Supplementary Fig. S7) of the products from trans-1,2-cyclopentanediol were less obvious. A total conversion of the two hydroxyl functions with the absence of the signal at δ (ppm) = 4.4 (m, 2 H, CH(OH)) was noticed. However, the sharpest singlet at δ (ppm) = 3.7 (s, 3 H, CH_3_O) was related to a methyl carbonate structure and the absence of signals at δ (ppm) = 5.4 (m, 2 H, CH-CH(O)) is relevant to the absence of cyclic carbonate. These results on model molecules confirm the initial hypothesis; the diol *cis* stereochemistry promotes cyclic carbonate based on esters exchange reaction. Then, the limiting step is the first dehydration leading to 3,6-sorbitan and 1,4-sorbitan. However, only 1,4-sorbitan presents the suitable stereochemistry to ensure five-membered cyclic carbonate formation.

### Catalyst screening and Sorb-BisCC kinetic study

Table 3 presents the results of a catalyst screening. In a first part, different tests were based on metallic catalysts since they are widely used on polymer synthesis and transesterification^49^. The results of these tests based on a transesterification between DMC and D-sorbitol with a distillation bridge, during 16 h are given in Table 3, Entries 2–4. No reaction occurs (yield of 0%) since the reaction temperature is too low to activated metallic catalysts, which are generally used in a temperature range of 150–250 °C^50,51^. Then, most efficient catalyst systems have been tested with two families based on strong bases associated with mineral (Table 3, Entries 5–7) or organic structures (Table 3, Entry 8–10). These latter can be divided into two main groups based on strong bases with (i) the phosphazene family, which are the strongest bases, and particularly BTPP which presents a pK_BH+_ = 28.89^52^ (Table 3, Entry 8), and (ii) the guanidine family with TBD and DBU, pK_BH+_ = 25.98 and 24.33, respectively (Table 3, Entries 9–10). BTPP gave similar results than DBU and TBD with a low yield, 20–25%. These results were surprising as it was expected that the strongest bases could increase the reaction rate and the yield. In fact, we could assume that the basicity of BTPP is too high and then promote side-reactions. In particular, it could be assumed that the carbonate rings are deprotonated to create an equilibrium between ring and linear-based carbonates. Table 3, Entries 5–7 present results with mineral bases. No reaction occurs with the strong mineral bases which have been also tested by Mazurek-Budzyńska and al^19^. They performed reactions with methanol as solvent and then obtain 41% of D-sorbitol conversion with K_2_CO_3_ as catalyst.

The use of a solvent should be taken into account to explain this difference of results, since the solvent can increase the mobility of the chemicals and the homogeneity of the medium. However, it is very well known that reactions without solvent present strong advantages for the environment and a green chemistry, for the scaling up, and on the final product cost. Based on these previous observations, the solubilities of the different catalysts at room temperature and 75 °C (i.e. the reactional temperature) were investigated. It appears that all the mineral catalysts were insoluble in DMC at both temperatures. However, TBD, DBU and BTPP were soluble in DMC at 75 °C. These solubility results confirmed the deprotonation of D-sorbitol in a tri-phase medium under ester-exchange conditions was impossible. Instead, when the catalyst is soluble in the reactional medium, its activity is promoted, and a solid/liquid reaction is then possible.

To conclude this screening part, strong organo-catalysts seem to be the most appropriate catalysts to perform this kind of reaction. This also highlights the fact that working in a green way i.e. in a reactive solvent with bi-phase medium implies an appropriate choice of catalyst. According to these previous results, organo-catalysts were selected after this screening step. More precisely, a system based on TBD was chosen since it presents a lower toxicity with similar yield results compared to DBU or BTPP.

**Figure 3:**
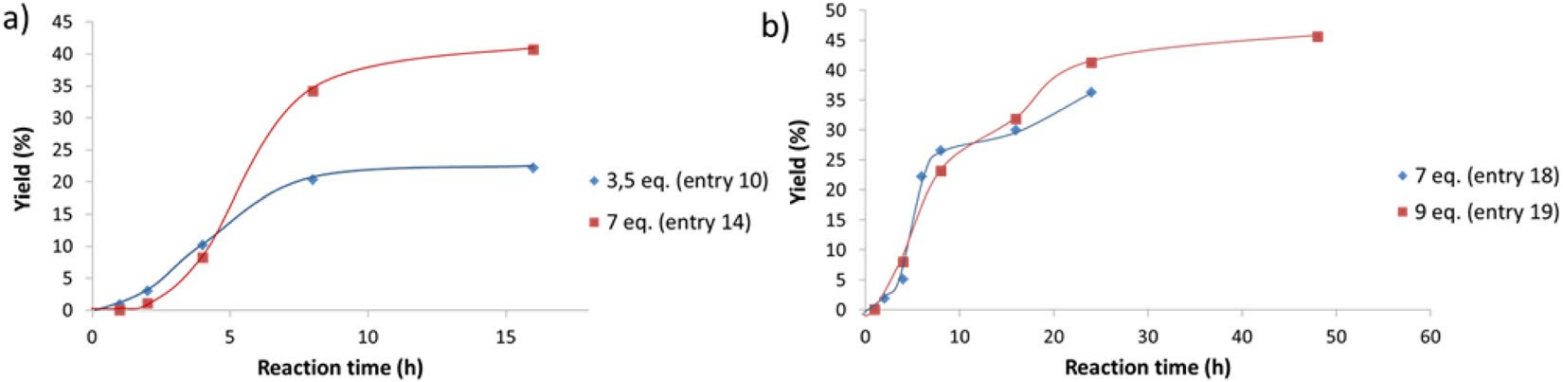
Influence of the number of equivalent of DMC on the kinetic profile with: (**a**) a distillation bridge device and (**b**) vigreux distillation column.

Figure 3 displays the results of kinetic evolutions with 3.5 and 7eq. of DMC toward sorbitol with a distillation bridge (Table 3, Entries 10 and 14).

With 3.5 eq. of DMC, a yield plateau at around 20% was reached after 8 h of reaction. With 7eq. of DMC, the same phenomenon occurs at 41%, also after around 8 h of reaction. These results were surprising considering the proposed reactional mechanism (Fig. 2), working with more than 3.5eq. of DMC equivalent is theoretically not needed. Supplementary Fig. S8 shows ^1^H-NMR analysis of the reaction distillate indicating DMC and methanol (MeOH) molecules in the medium. It pointed out the formation of an azeotrope between DMC and MeOH. Such an azeotrope has been previously reported^53^ with a DMC/MeOH molar ratio close to 30/70. Additionally, the evaporation of DMC by vapor pressure cannot be excluded.

The effect of different experimental conditions and devices were investigated to increase the reaction yield and to compensate the potential loss of reactant. Optimum results were 40% yield with 5 eq. of DMC and 10% yield with distillation bridge (Table 3, entry 13) and reflux device (Table 3, entry 15), respectively. Finally, with vigreux fractionating column, several syntheses were performed with various content of DMC. The main kinetic results of the corresponding study are presented on Fig. 3. The profiles are similar to the previous one and present a plateau at 24–48 h of reaction. Nevertheless, the option of breaking the azeotrope with a vigreux fractionating column cannot be selected. Indeed, a remaining amount of DMC is still distilled with the methanol and the reaction was not shifted in favor of the synthesis of Sorb-BisCC. The global yield only increases of 5% with a 32 h long reaction compared to the obtained result with a distillation bridge. More detailed information is available in the supporting information (section S.0.3). All results are summarized in Table 3.

This kinetic study shows that the optimum yield at low reactional temperature was 40% with 5eq. of DMC, TBD as catalysts with a distillation bridge device over 16 h reaction. However, a maximum yield of 45% can be reached using a vigreux fractionating column during 48 h. This reactional time is three times superior than those performed with a distillation bridge. These results are promising since the formation of 1,4 sorbitan with 3,6 sorbitan cannot be avoided, limiting consequently the yield, according to the mechanism proposed on Fig. 2.

### Sorb-BisCC yield optimization based on continuous flow rate system

To optimize the DMC intake and increase the conversion of D-sorbitol into Sorb-BisCC; another reactional setup with a continuous feeding of DMC was incremented. The DMC feed was regulated at 0.3 mL/h (Table 4, Entry 2–3) and initial DMC/D-sorbitol (mole/mole) ratios were fixed at 1 (Table 4, Entry 3) and 3.5 (Table 4, Entry 2). Reactions yield were 35 and 43%, respectively. Compared to previous results, the yield increase was not significant in these conditions. When initial DMC/D-sorbitol ratio was set to 1, the low content of reactive solvent prevents a correct homogenization of the medium and the global reactivity of the system is decreased. Then, with the ratio set to 3.35, the conditions are very similar to the optimum one with a distillation bridge. It is thus coherent to obtain a similar reaction yield.

According to these observations, reactional settings were adjusted. The DMC/D-sorbitol ratio was maintained at 3.5, but the DMC flow rate was decreased to 0.15 mL/h to maintain the reactant content as low as possible in the reactional medium in order to minimize reactant loss by vapor tensor and maintain the azeotrope formation. In this case, a total of 8.5 eq. of DMC in respect to the D-sorbitol were used i.e. an excess of 5.5 eq. Similar conditions were used for the reaction presented in Table 3, entry 19 with a classical reaction setup, leading to 45% yield in 48 h. Instead the use of continuous feeding of DMC successfully increased the yield to 50% in 16 h Table 4, entry 1).

### Synthesis of polyether/polycarbonate by ROP of Sorb-BisCC

**Figure 4:**
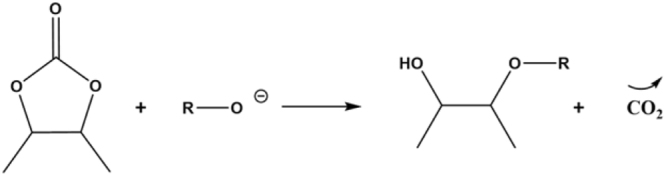
General mechanism to obtain ether linkage from five-membered BisC, with CO_2_ formation.

The conditions of reactions between 1-octanol and Sorb-BisCC are presented in Table 5. A gas release was observed for all reactions on agreement with the mechanism presented in Fig. 4^54^.

**Figure 5:**
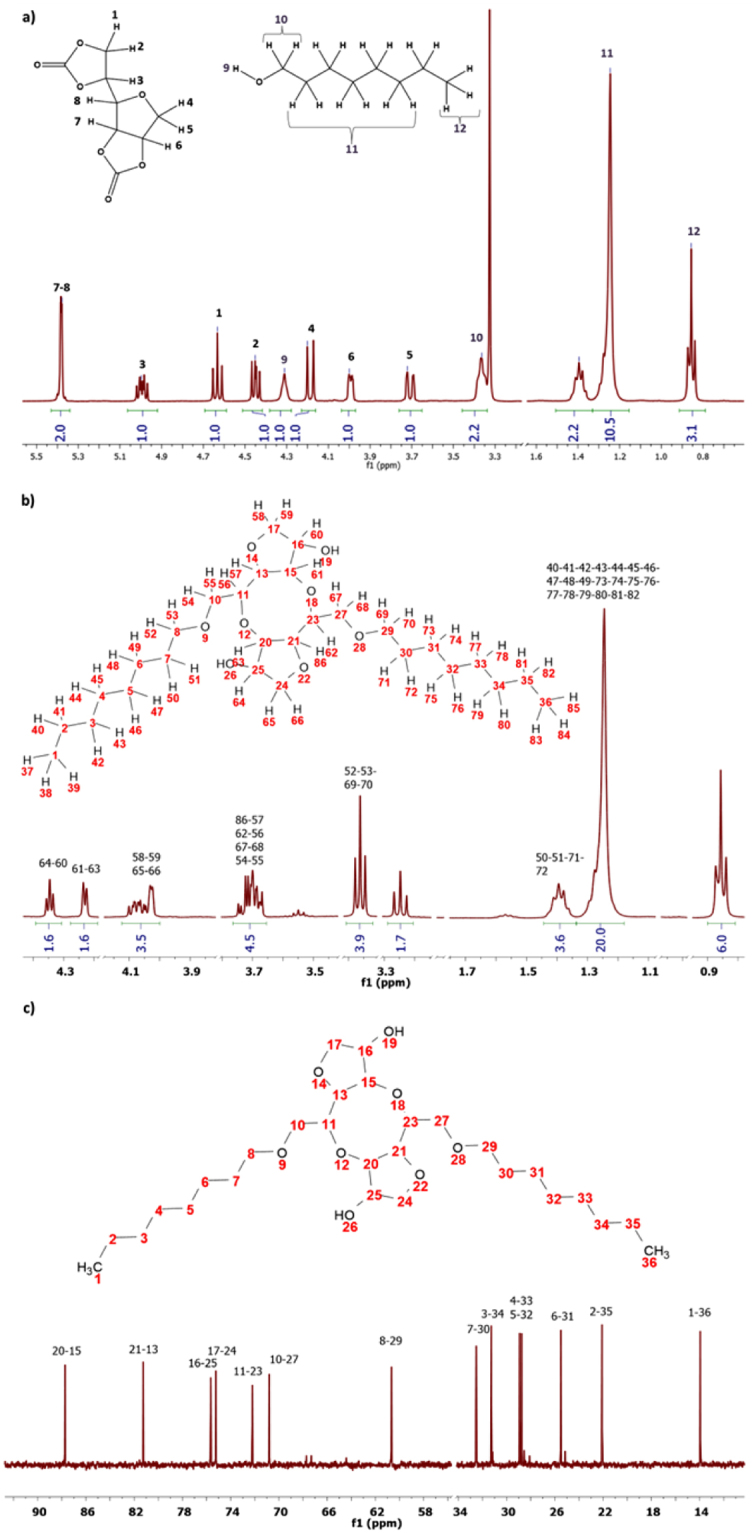
NMR spectra: (**a**) ^1^H-NMR of the Sorb-BisCC and 1-octanol mixture (**b**) ^1^H-NMR and (**c**) ^13^C-NMR of the synthesized product.

Table 5 Entry 1 corresponds to a model reaction between an alcohol initiator and Sorb-BisCC to control the reactivity of the five-membered carbonate cycles. After two hours of reaction, the medium was directly analyzed by ^1^H-NMR (Fig. 5b), ^13^C-NMR (Fig. 5c) since the reaction was performed in DMSO-d_6_. Results were compared to the initial medium (before the reaction starting) (Fig. 5a). The full consumption of the five-membered carbonate and hydroxyls groups was controlled through the absence of the peak at δ (ppm): 5.4 (m, 2 H, CH-CH(O)) and δ (ppm): 4.3 (s, 1 H, CH_2_-OH)). Sorb-BisCC is highly reactive in these conditions and released hydroxyl groups from the ROP were able to react on another Sorb-BisCC to coupled two initiated Sorb-BisCC. The resulting coupled molecule is a diol and is presented in Fig. 5b,c. Thus, a Sorb-BisCC molecule can act as cross-linking point, once the two cyclic(carbonate) are opened, two hydroxyls groups are released and available to initiate another Sorb-BisCC molecule.

Supplementary Fig. S9 (1), (2) and (3) present the three main molecules which can be theoretically obtained by this reaction. According to ^13^C-NMR spectrum (Fig. 5c), the reactional product is 5,10-bis((octyloxy)methyl)octahydro-2*H*,5*H*-difuro[*3,2-b:3′,2′-f*][1,5]dioxocane-3,8-diol (Supplementary Fig. S9 (3)).

Anionic ROP were conducted with 10 to 100 eq. of Sorb-BisCC, in respect to the 1-octanol, with 0.7% mol of catalyst (Table 5, Entries 6–9). Brown viscous products were obtained, (Table 5, Entries 6 and 7), and sticky brown materials (Table 5, Entries 8 and 9). SEC analyzes (Supplementary Fig. S10) show two main oligomers populations with degree of polymerization (DPn) of 5 and 10 (Table 8), respectively (determined from Equation (1)). When 10 eq. of initiator were used (Entry 9), only one population was detected with a DPn = 5 (Table 8). Similar DPn were observed when the reaction was conducted with 25 or 100 eq. of Sorb-BisCC. (Table 8, Entry 6, 8). Consequently, 25 eq. of Sorb-BisCC seems to be an optimum. A low molar masses peak (i.e. 360 g/mol) was sometimes observed, corresponding to residual Sorb-BisCC.

**Table 8:** Properties of main synthetized macromolecular architectures.

Entry	Mn_1_ (g/mol)	Mn_2_ (g/mol)	Mn_3_ (g/mol)	DPn	I-OH content (mmol/g)	II-OH content (mmol/g)	Global OH value (mg KOH/g)	T_deg 5%_	T_deg50%_	Chars (% wt)
2	*Insoluble*							140	440	0
3								98	200	25
4								92	271	35
5	1590	n.o.	n.o.	7	0.6	4.6	288	114	196	0
6	340	1360	2380	10	0.9	4.1	276	138	258	0
7	340	1340	2315	10	0.7	4.2	273	90	228	2
8	1390	2500	n.o.	10	0.8	4.7	305	125	248	8
9	260	1280	n.o	5	0.9	3.6	250	119	244	9

n.o.: not observed

According to the proposed opening mechanism (Fig. 4), primary and secondary hydroxyl groups can be obtained. Each time, a five-membered cyclic carbonate is opened. The hydroxyl contents of each oligomer were determined by quantitative ^31^P-NMR (Supplementary Fig. S11) and show a low content in primary hydroxyl groups. The global OH value is comprised between 4.5 and 5.5 mmol/g (Table 8).

From the synthesis presented in Table 5, Entries 2–4 present a higher content in catalyst in order to increase the reaction rate. In each case, a solidified and brittle brown material was obtained. Supplementary Fig. S12 shows FTIR analysis of the corresponding materials. The absence of the characteristic peak at 1778 cm^−1^ (C=O, five-membered cyclic carbonates) and the large signal at 3200 cm^−1^ corresponding to O-H elongation are strong indications that the full conversion of the Sorb-BisCC occurs. The presence of the bands at 1680 and 1040 cm^−1^ are related to C=O stretching of the linear carbonate and the C-O stretching of ethers linkage^55^, respectively. Nevertheless, these three different products were insoluble in most of the common solvents even after acetylation due to the cross-linked architectures. Then, the NMR quantification of ether and carbonate linkage contents were impossible to be correctly performed.

### Synthesis of polyhydroxyurethanes

Four PHU were synthetized from different short diamines (C4 to C6) and DDA (C36) (Table 6, Entries 1–4). Then, two families of PHU were synthetized from a blend of DDA and short diamines to investigate the effect of the fatty diamines on the chemical and thermal properties of the resulting PHU (Table 6, Entries 5–12).

**Figure 6:**
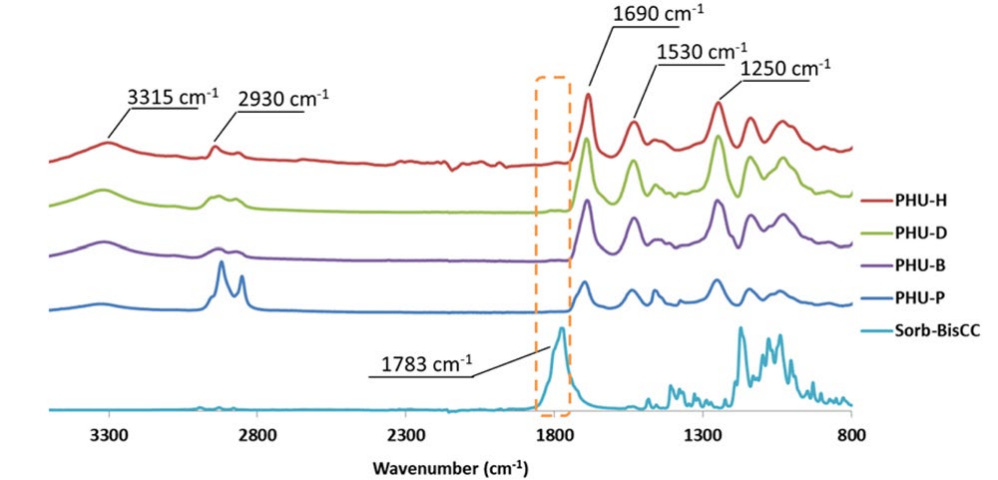
FT-IR spectra of Sorb-BisCC, PHU-P, PHU-B, PHU-D, PHU-D and PUH-H.

**Figure 7:**
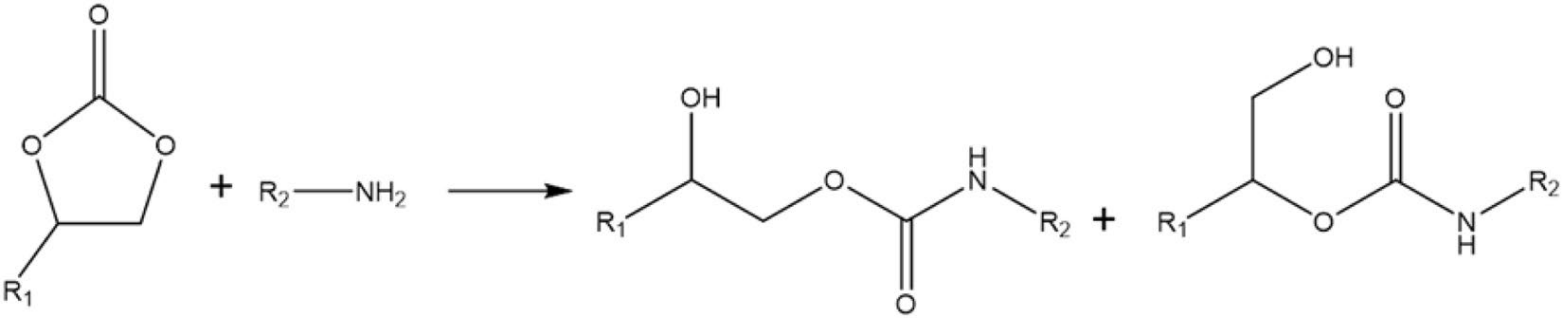
Aminolysis reactional pathway between a five-membered BisCC and a primary amine.

Figure 6 presents the FTIR spectra of the initial monomer (Sorb-BisCC) and PHU-P, PHU-B, PHU-D and PHU-H. Sorb-BisCC presents, as previously described, a characteristic peak at 1783 cm^−1^ corresponding to the carbonyl bond. This peak is missing for the different synthetized PHUs showing the full consumption of Sorb-BisCC. All PHUs present the distinctive bands of the urethane groups located at 3300–3400 cm^−1^ (–NH stretching merge with the –OH stretching), 1690 cm^−1^, 1530 cm^−1^ (-NH, bending) and 1250 cm^−1^ (=CO, stretching)^28^. The O-H stretching band at 3300–3400 cm^−1^ is due to the aminolysis reaction presented in Fig. 7, on perfect agreement with the expected chemical structure of PHUs. A shoulder or second peak located around 1650 cm^−1^ is characteristic of amide formation. This peak is absent since the monomers do not present ester linkages for PHU-B, PHU-D, PHU-H and PHU-P^38^. The PHU obtained from the diamines blend present similar FT-IR spectra (Supplementary Fig. S13).

Table 6 presents the number-average molar mass (Mn) of the synthetized PHUs. All SEC curves are available in Supplementary Figs S14 and S15. Mn values are rather low, comprise between 1450 and 2430 g.mol^−1^ for all PHUs. However, it is well known that PHUs generally present low Mn and high Đ (Table 6) due to different factors such as a wide number of side reactions^56^. The molar masses obtained in this study are comparable to other published NIPU systems^37,57^.

The PHUs OH-values presented in Table 6 evolve from around 9 mmol/g for PHU-B, PHU-D, PHU-H to 3 mmol/g for PHU-P, in full agreement with the aminolysis reaction, presented on Fig. 7.

It is obvious that hydroxyl groups content decrease with the diamine chain length increase. Then the PHUs functionality can be tuned. Indeed, the diamine blend strategy provides a wide range of OH-values. These hydroxyl groups can be used for PHU crosslinking to modify the properties by increasing the molar masses of the PHUs.

Supplementary Figs SI16–19 and S20–23 present the ^1^H-NMR and ^13^C-NMR spectra of PHU-P, PHU-B, PHU-D, PHU-H, respectively, with the main peaks attribution. These NMR spectra confirm the macromolecular architectures of the PHUs on agreement with previous FT-IR observations.

**Figure 8:**
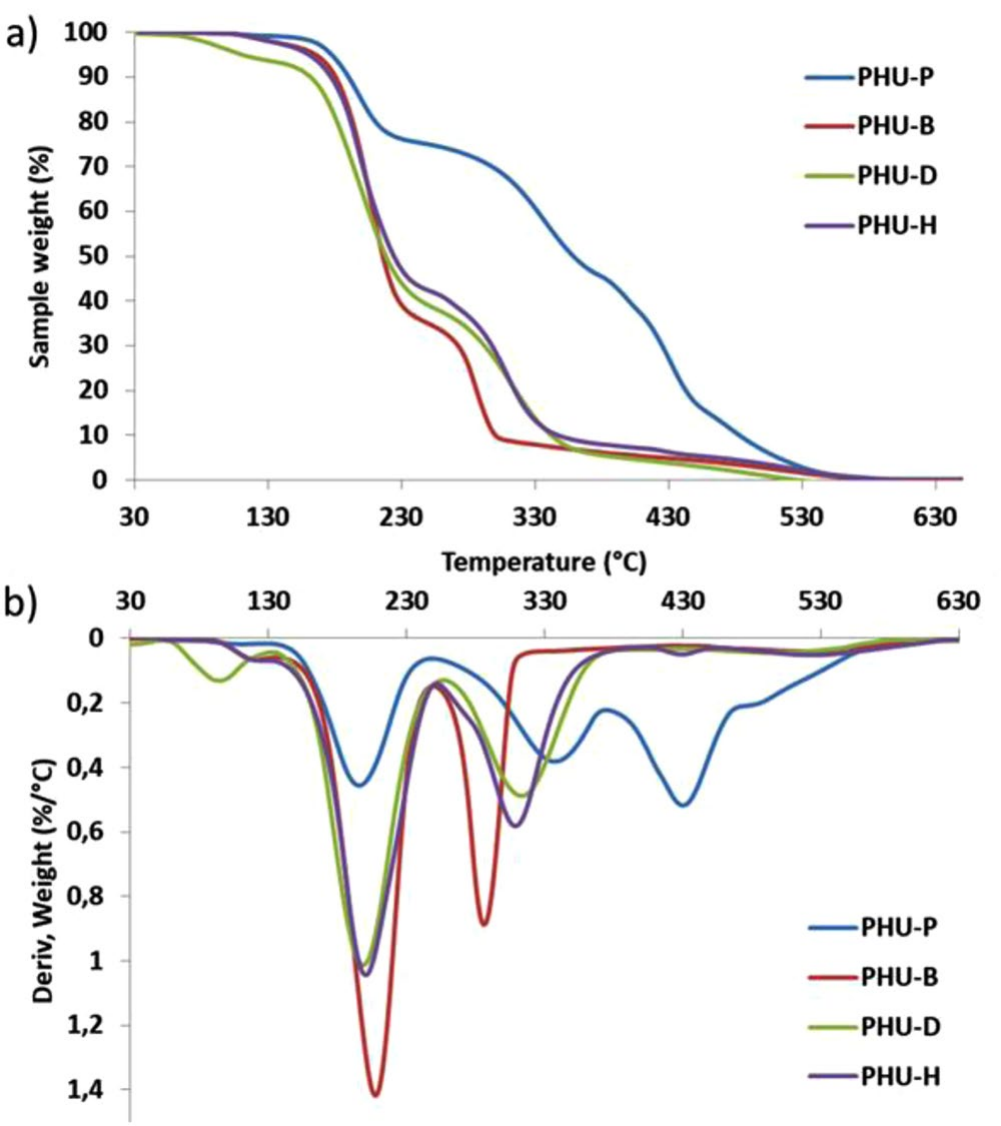
(**a**) TGA and (**b**) DTG curves of PHU-P, PHU-B, PHU-D and PHU-H.

TGA and DTG curves of PHU-P (Fig. 8a,b) present a three-step weight loss. This complex thermal degradation is linked to the urethane function reversibility within a temperature range from 120 to 250 °C depending of the neighbor groups^58^. At higher temperature, irreversible degradation mechanism of the urethane bond followed by carbon-carbon bonds cleavages take place. However, PHUs obtained from shorter diamines present a two-stage weight loss based on urethane function reversible and irreversible degradation mechanism. The resulting low molar masses PHUs fragments, linked to the short monomers compared to DDA, are vaporized before that carbon-carbon bonds cleavages could occur. As previously described^59^, PHUs with the highest urethane bonds content present the lower thermal stability such as PHU-B or PHU-H, for instance compared to PHU-P. PHU-H presents the lowest thermally stability, certainly due to the presence of methyl groups along the backbone due to the chemical structure of 1.5-diamino-2-methylpentane. It is very well known that the methyl group is electron donor which promotes the urethane degradation into primary amines and olefins^59^.

Finally, PHU obtained from diamines blends presents weight loss profiles comprised between the PHU-P profile and the PHU-B or the PHU-D profile, as shown in Supplementary Fig. S24. The characteristic temperature at 50% of weight loss (T_deg50%_) ranges between 216 and 318 °C for PHUs based on 1.4 B (Table 6). T_deg50%_ of PHU based on 1.5 MD evolved from 217 to 304 °C (Table 6). As previously described, the increase of the DDA content in the macromolecular architecture, decreases the global thermal instability linked to the urethane bonds.

TGA analyses have shown that no major degradation occurs during the the studied temperature range of the DSC analysis. Tg of the PHUs are presented in Table 6. PHU-B, PHU-D and PHU-H have similar Tg (around 41 °C), whereas PHU-P presents a Tg below 0 °C. This low Tg is linked with DDA building block and its pending chains since they bring chains mobility and a Tg decrease^60–62^. As previously shown on thermal properties, the diamine blending can allow to tune the Tg of the corresponding PHU. As presented in Supplementary Fig. S25, Tg from PHU-0.2B to PHU-0.8B and from PHU-0.2D to PHU-0.8D gradually evolved from below zero to room temperature (Table 6) as a function of the DDA content and evolved according to a polynomial fit presented in SI (Equations S.2 and S.3).

## Conclusions

The Sorb-BisCC, a promising biobased platform molecule, was successfully synthetized from D-sorbitol in eco-friendly condition i.e. low temperatures, catalyzed reactions, reactive solvents and renewable reactants. A deep study of the reactional conditions and setups was conduct to determine the optimum pathway resulting in a strong increase of the synthesis yield (+25%) compared to previous works based on green reactions. One of the key parameter was the catalyst solubility in DMC, the reactive solvent. The most fruitful results were obtained with TBD as catalyst and a continuous feed of DMC into the reactional medium. To show that Sorb-BisCC is a platform molecule, it was used as a basis leading to the elaboration of different products with specific architectures: (i) a diol (ii) crosslinked polyethers and (iii) PHUs. All Sorb-BisCC derivate present non-conventional molecular architectures from the fused tetrahydrofuran ring, with the resulting hydroxyl groups obtained from the rings opening. In the case of polyether’s synthesis, these hydroxyl groups show a reactivity high enough to polymerize with another Sorb-BisCC to form a chain. This platform molecule was also involved in PHUs synthesis with different short and fatty diamines. The chain length of the diamine was an effective leverage on PHUs glass transition. The resulting non-isocyanate polyurethanes present a large range of glass transition temperatures from −9 to 42 °C.

To conclude, Sorb-BisCC has proved to be an efficient biobased platform molecule, leading to a large range of products, using different chemical pathways, with a large portfolio of properties.

## Electronic supplementary material

Supporting Information
