# Supplementary material for: From D-sorbitol to five-membered bis(cyclo-carbonate) as a platform molecule for the synthesis of different original biobased chemicals and polymers
Source: Sci Rep. 2018 Jun 14;8:9134. doi: 10.1038/s41598-018-27450-w (PMC6002542; doi:10.1038/s41598-018-27450-w)
Supplement: Supplementary file 1 — Supporting Information [file 41598_2018_27450_MOESM1_ESM.docx]

**Supporting information**

**From D-sorbitol to five-membered bis(cyclo-carbonate) as a platform molecule for the synthesis of different original biobased chemicals and polymers.**

Pierre Furtwengler^a^, Luc Avérous^a^

*^a^BioTeam/ICPEES-ECPM, UMR CNRS 7515,*

*Université de Strasbourg, 25 rue Becquerel, 67087 Strasbourg, Cedex 2, France*

Table of content

[S.1. Sorb-BisCC analysis 2](#_Toc514138603)

[S.2. ^1^H-NMR spectra 4](#_Toc514138604)

[S.3. Relationship between Sorb-BisCC yield and the reactional device 7](#_Toc514138605)

[S.4. Study of the potential products obtained from the reaction between Sorb-BisCC and 1,8-octanediol 8](#_Toc514138606)

[S.5. SEC analysis 9](#_Toc514138607)

[S.6. ^31^P-NMR of ROP products between D-sorbitol and 1,8-octanol 10](#_Toc514138608)

[S.7. FTIR of polyether/polycarbonate synthetized from Sorb-BisCC 12](#_Toc514138609)

[S.8. Analysis of the PHUs 13](#_Toc514138610)

[a. FTIR analyses 13](#_Toc514138611)

[b. SEC analyses 14](#_Toc514138612)

[c. ^1^H-NMR spectra 16](#_Toc514138613)

[d. ^13^C-NMR spectra 18](#_Toc514138614)

[e. ATG and DSC analysis of PHUs samples 20](#_Toc514138615)

# Sorb-BisCC analysis

This section is dedicated to Sorb-BisCC characterization. Fig S. 1-2 present the FT-IR spectrum and DSC thermogram of the synthesized Sorb-BisCC. Fig S. 3-4 show ^1^H-NMR and ^13^C-NMR spectra with the signals attribution linked with the chemical structure.


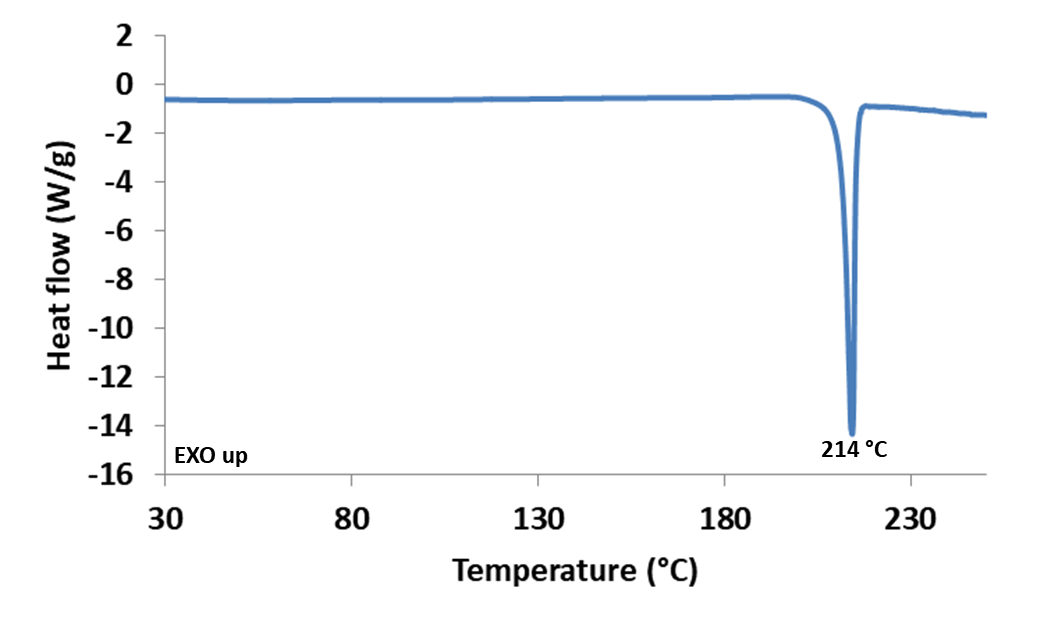


Fig SI 1: DSC analysis of Sorb-BisCC


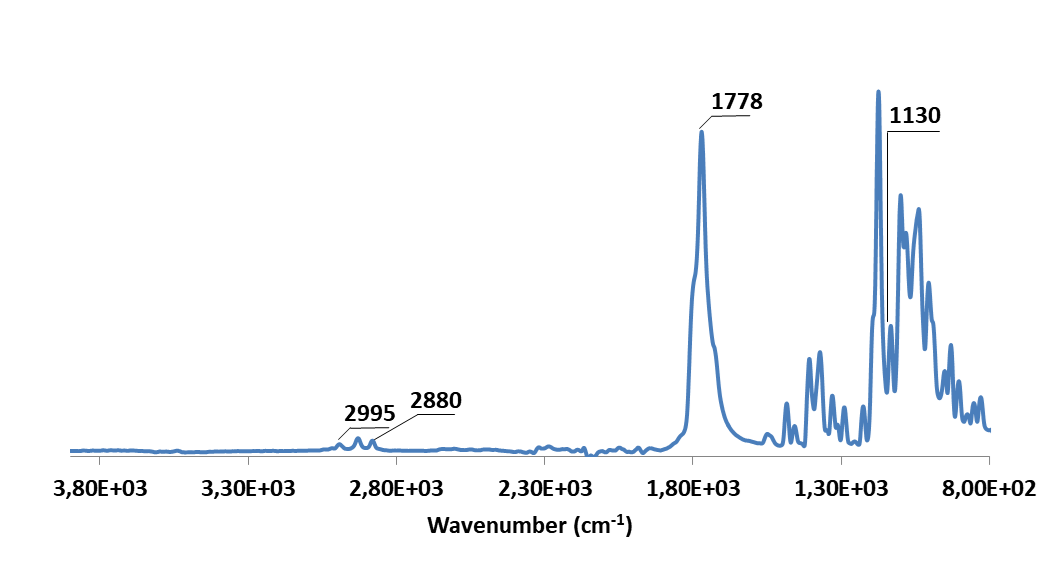


Fig S. 2: FT-IR analysis of Sorb-BisCC


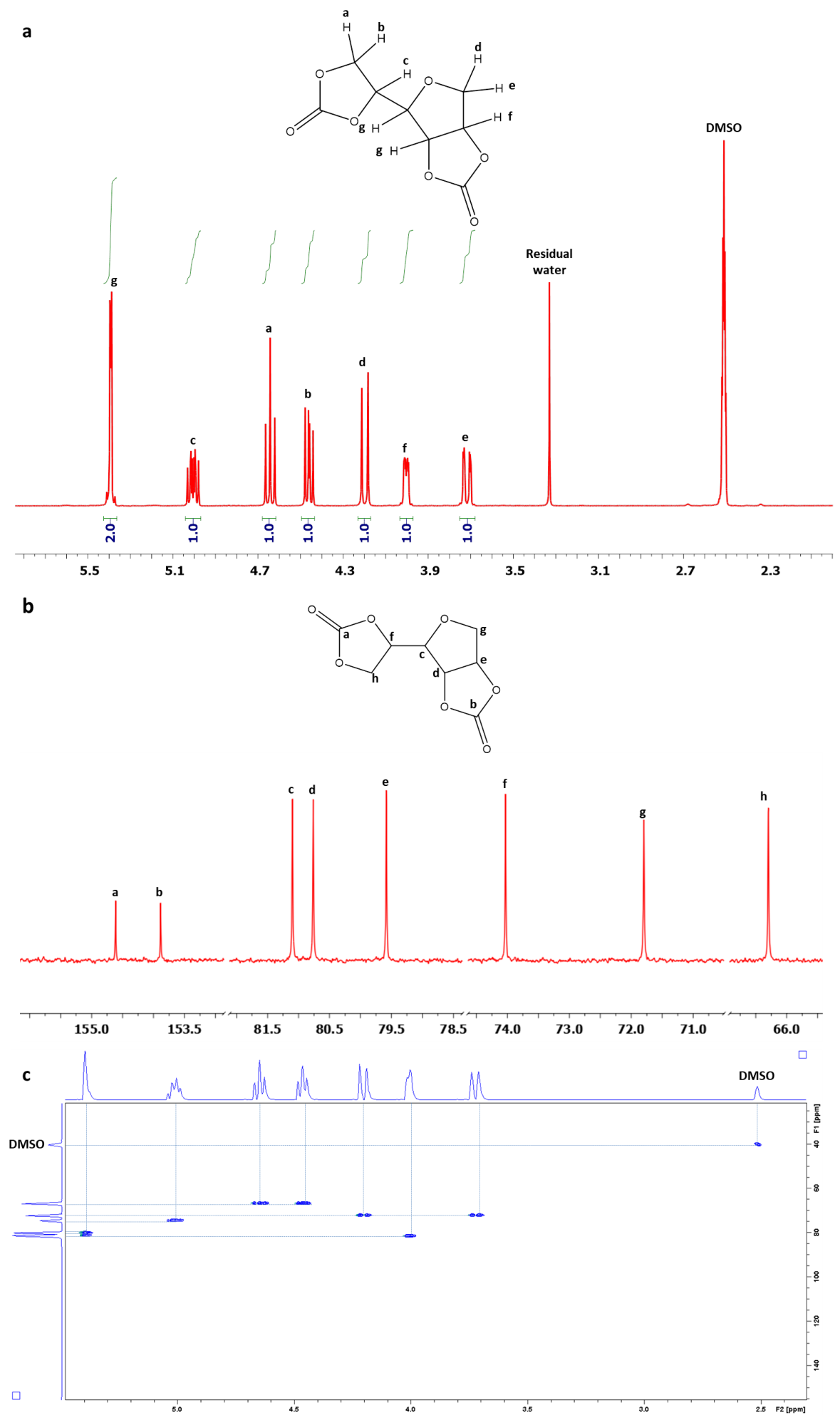


Fig S. 3 (a) ^1^H-NMR spectrum, (b) ^13^C-NMR spectrum and (c) HSQC-NMR spectrum of Sorb-BisCC

# ^1^H-NMR spectra

This section is dedicated to ^1^H- NMR spectra of cis-1,2-cyclopentanediol, (±)-trans-1,2-cyclopentanediol and the products obtained after reaction with DMC and TBD. ^1^H- NMR spectrum of the distillated product from the reaction between sorbitol and DMC (TBD catalyzed) is also available.


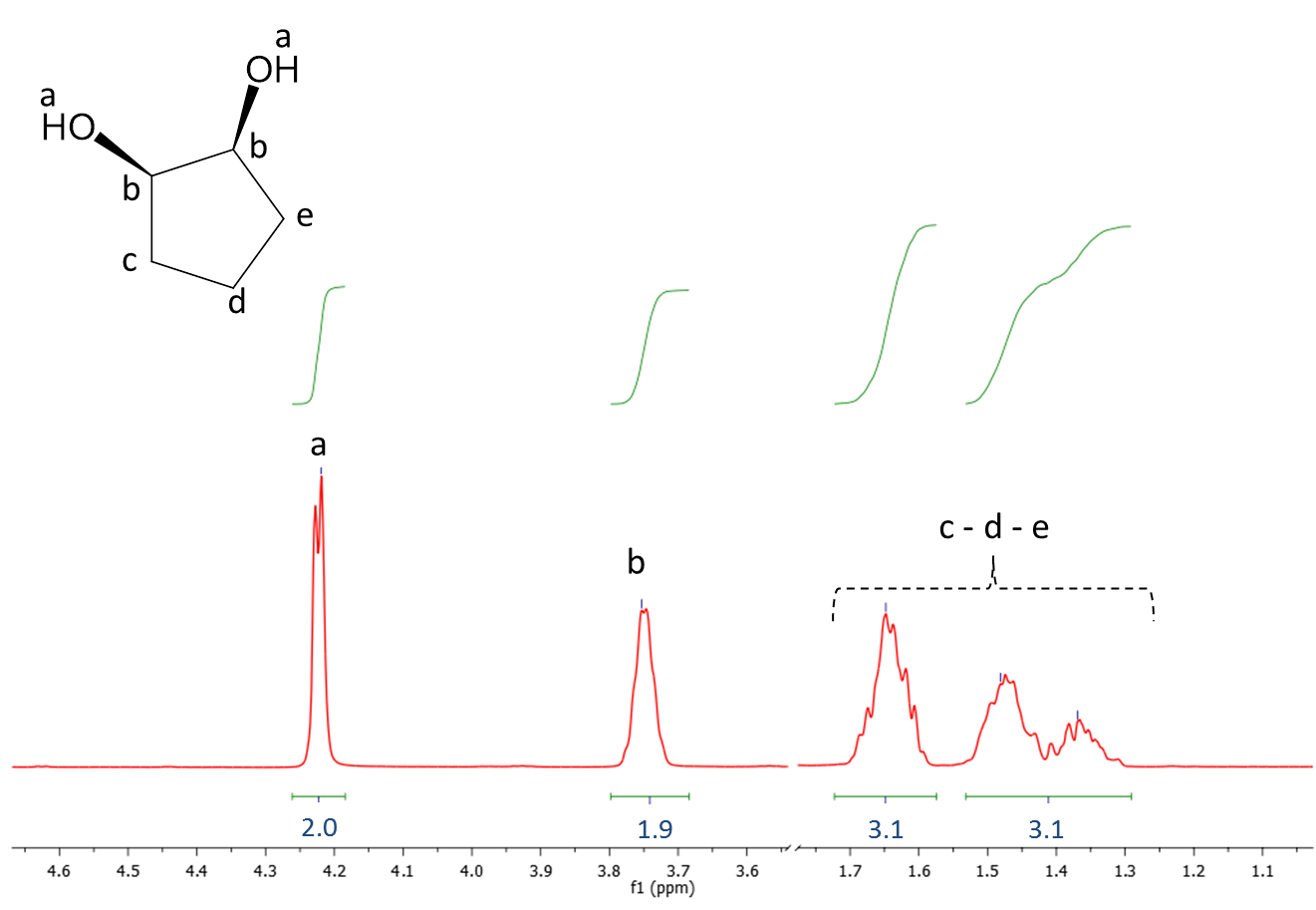


Fig S. 4: ^1^H-NMR cis-1,2-cyclopentanediol before the reaction with dimethyl carbonate


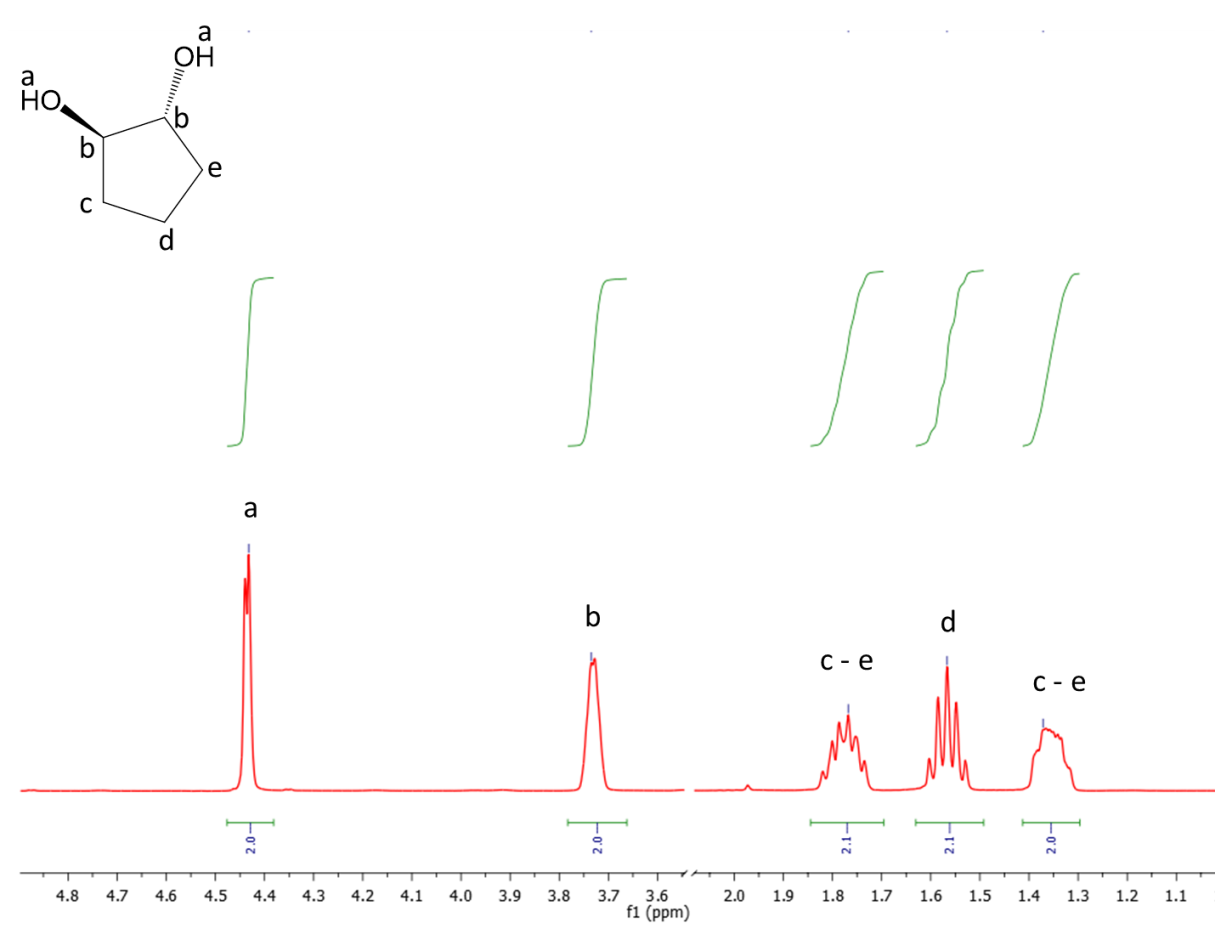


Fig S. 5: ^1^H-NMR trans-1,2-cyclopentanediol before the reaction with DMC


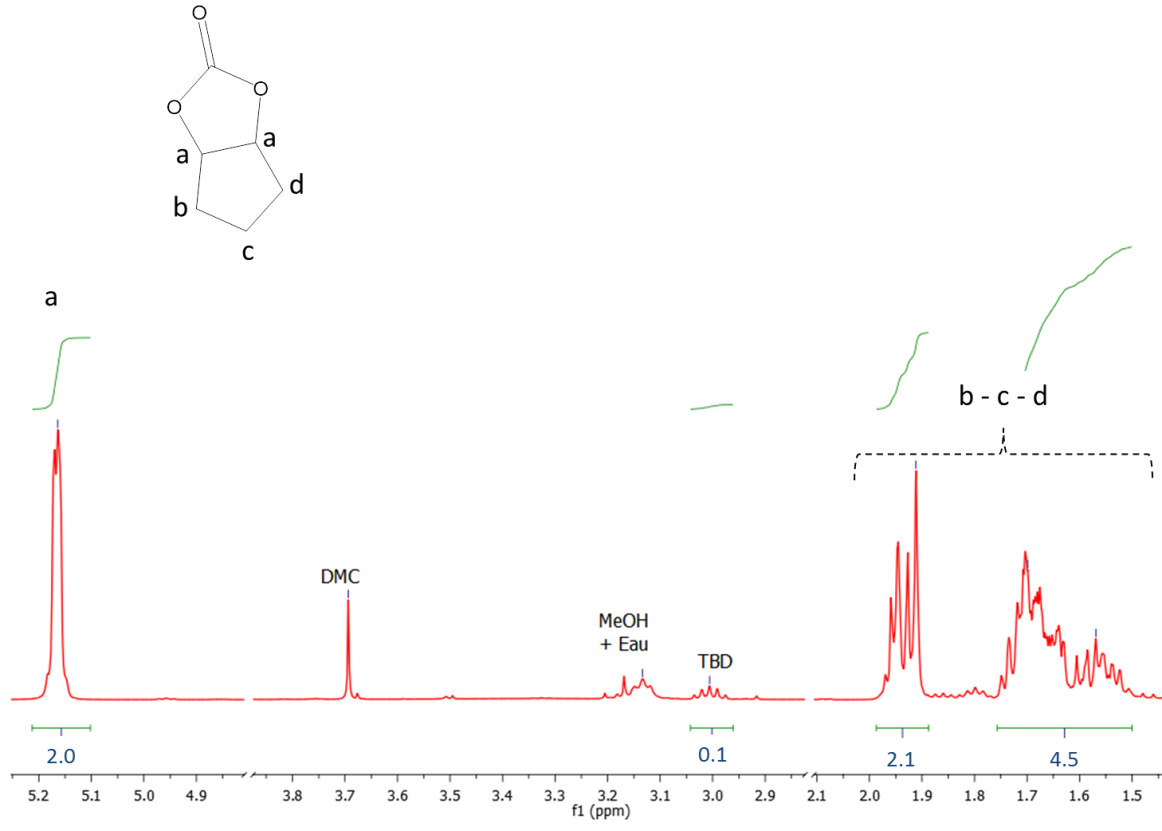


Fig S. 6: ^1^H-NMR cis-1,2-cyclopentanediol after the reaction with DMC


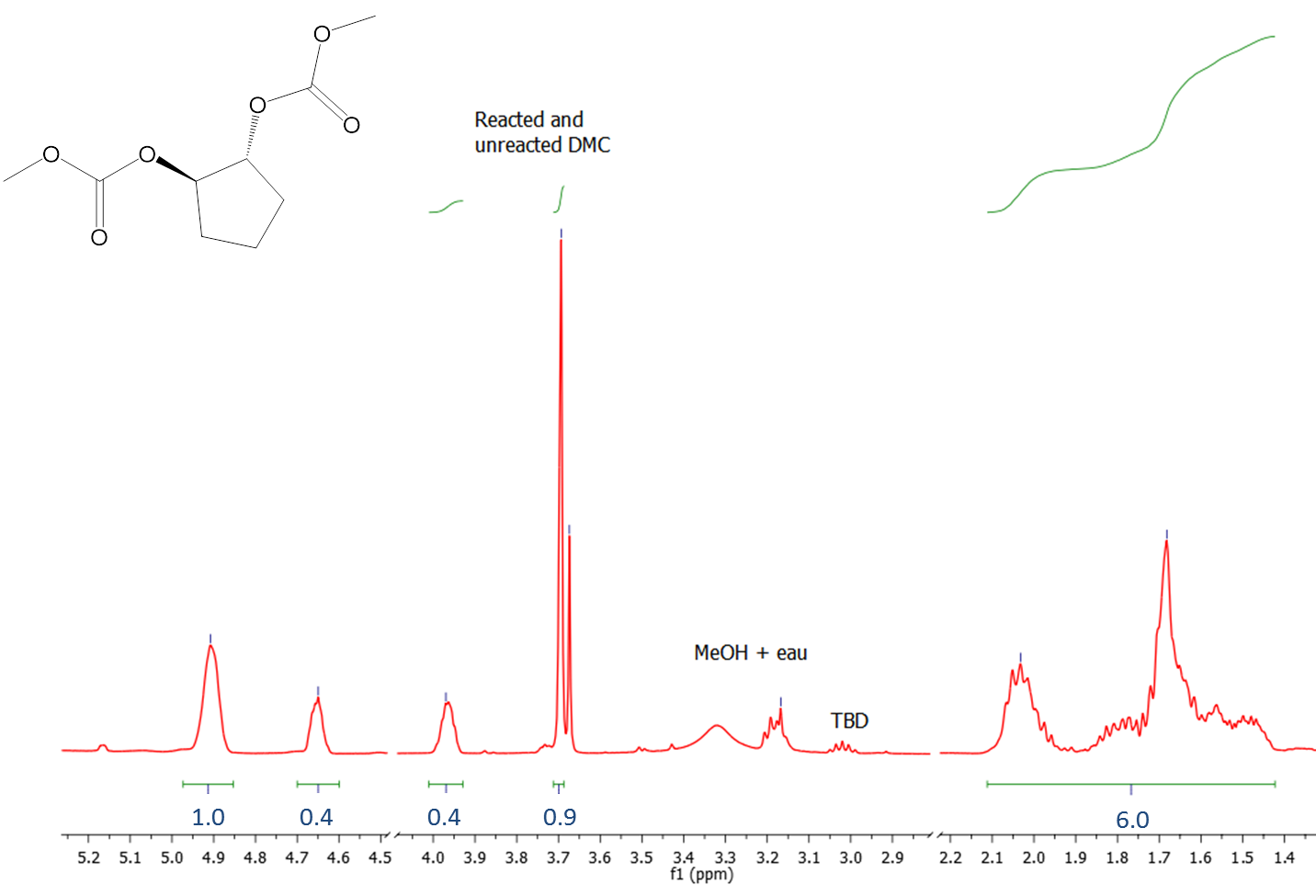


Fig S. 7: ^1^H-NMR trans-1,2-cyclopentanediol after the reaction with DMC

Fig S. 8: ^1^H-NMR of distillated subsidiary products of the catalyzed reaction between D-sorbitol and DMC

# Relationship between Sorb-BisCC yield and the reactional device

To compensate the effect of DMC loss with the azeotrope, two different options can be developed:

(i) The first option was to increase the initial quantity of DMC. Nevertheless, 5eq. of DMC (Table 3, entry 13) seems to give a yield optimum (40%).

(ii) The second option was to use a reflux system to condensate the evaporated reactant or a vigreux fractionating column to break the azeotrope. Both options were studied (Table 3, Entry 15-16).

It appears that reflux device was not suitable as the yield is low (10% against 15% with the vigreux fractionating column). In similar condition with distillation bridge (Table 3, Entry 10), a yield of 22% was obtained. With the vigreux fractionating column, the number of equivalents of DMC was increased to 5, 7 and 9eq., respectively (Table 3, Entries 17-19). The reaction time was increased up to 48 h with 9eq. DMC. Final corresponding yields were 14, 36 and 45%, respectively. Reactions were pushed to 12 and 18 eq. of DMC and a distillation vigreux columns (Table 3, Entries 20-22). However, the increased of DMC yield in high amount of by-products. The recovered Sorb-BisCC was not pure after the recovery in water. The main explanation linked to this result was that the high availability of DMC in the media promotes the synthesis of ‘’branched D-sorbitol’’ with linear carbonates instead of carbonate cyclization reactions.

# Study of the potential products obtained from the reaction between Sorb-BisCC and 1,8-octanediol


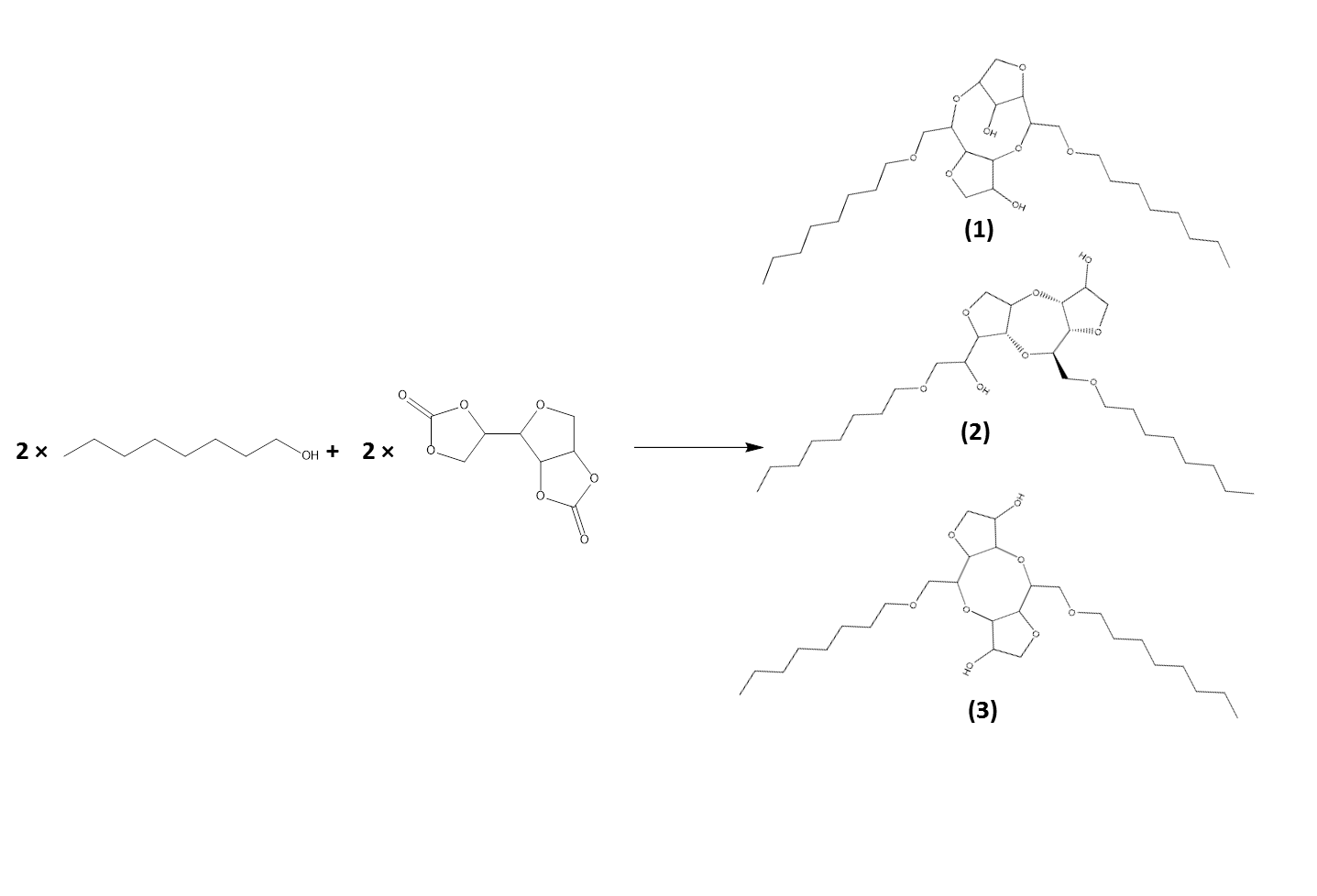


Fig S. 9: Theoretical reactional product one mole of Sorb-BisCC with two moles of 1-octanol.

# SEC analysis

The SEC analyses of the soluble macromolecules after an acetylation step are presented in this section.


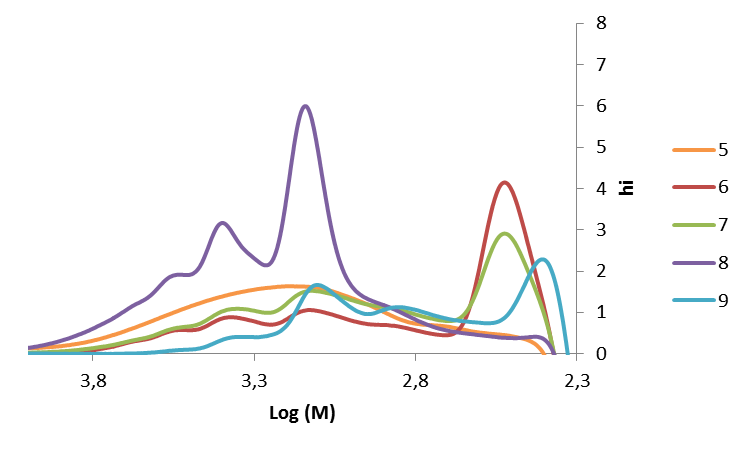


Fig S. 10: SEC curves of macromolecules products Entries 5 to 9 (Table 6)

# ^31^P-**NMR of ROP products between D-sorbitol and 1,8-octanol**

The targeted areas of ^31^P-NMR spectrum were based on the signal at 132.2 ppm corresponding to environmental water, the standards signal at 144.85 ppm (cholesterol, 1 mol/L), carboxylic acid area at 134-135.5 ppm and hydroxyl area at 149-141.5 ppm. We also notice a small unexpected peak at 140.6 ppm, which comes (after several investigations) from the molecular sieves used for the solvent storage.

Fig S. 11 presents ^31^P-NMR spectra of oligomer Entries 5 to 9 (Table 8) with the integration of the characteristic signals for quantification.


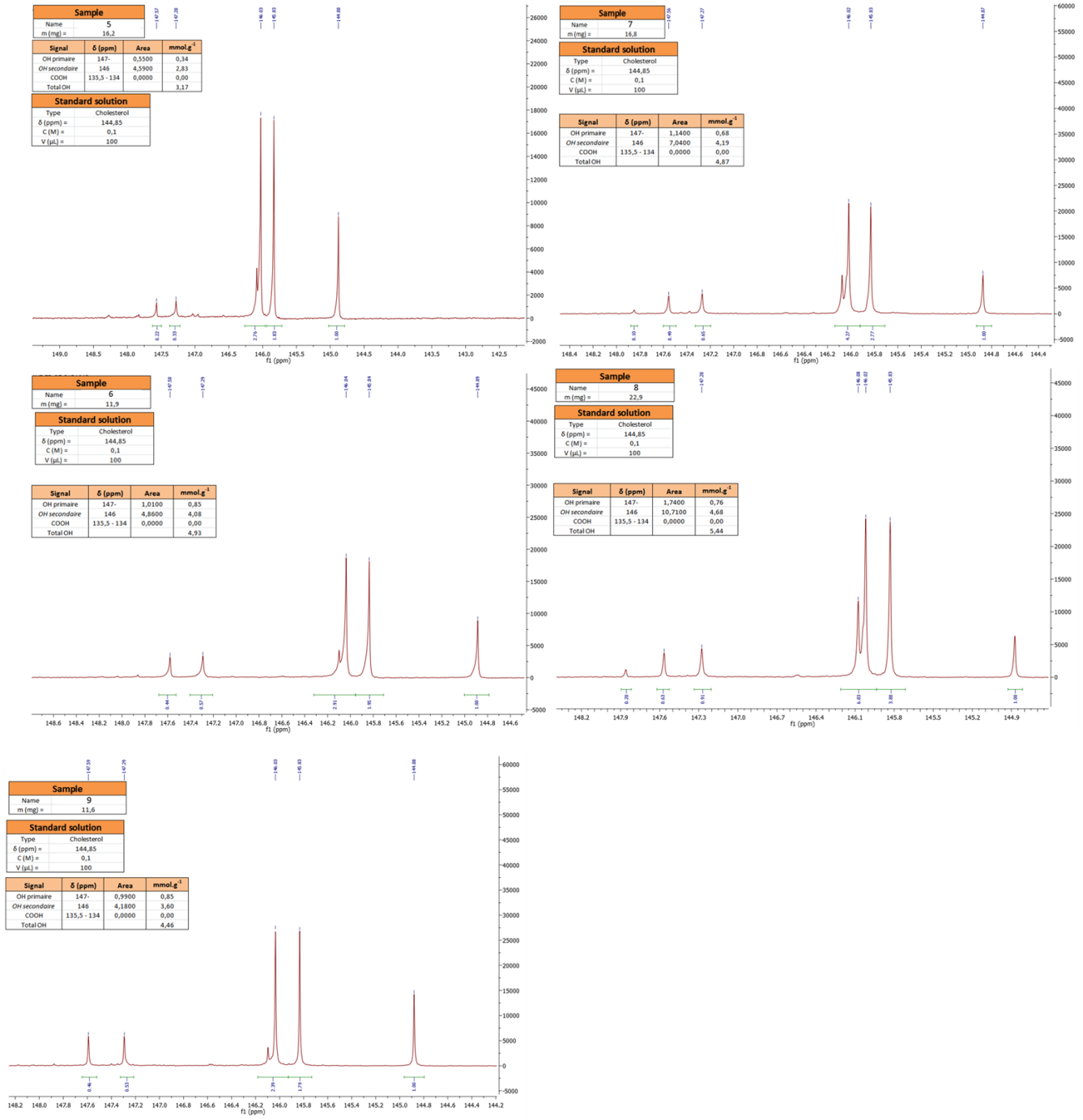


Fig S. 11: ^31^P-NMR spectra and hydroxyl content of macromolecules 5 to 9 from Table 8

# **FTIR of polyether/polycarbonate synthetized from Sorb-BisCC**


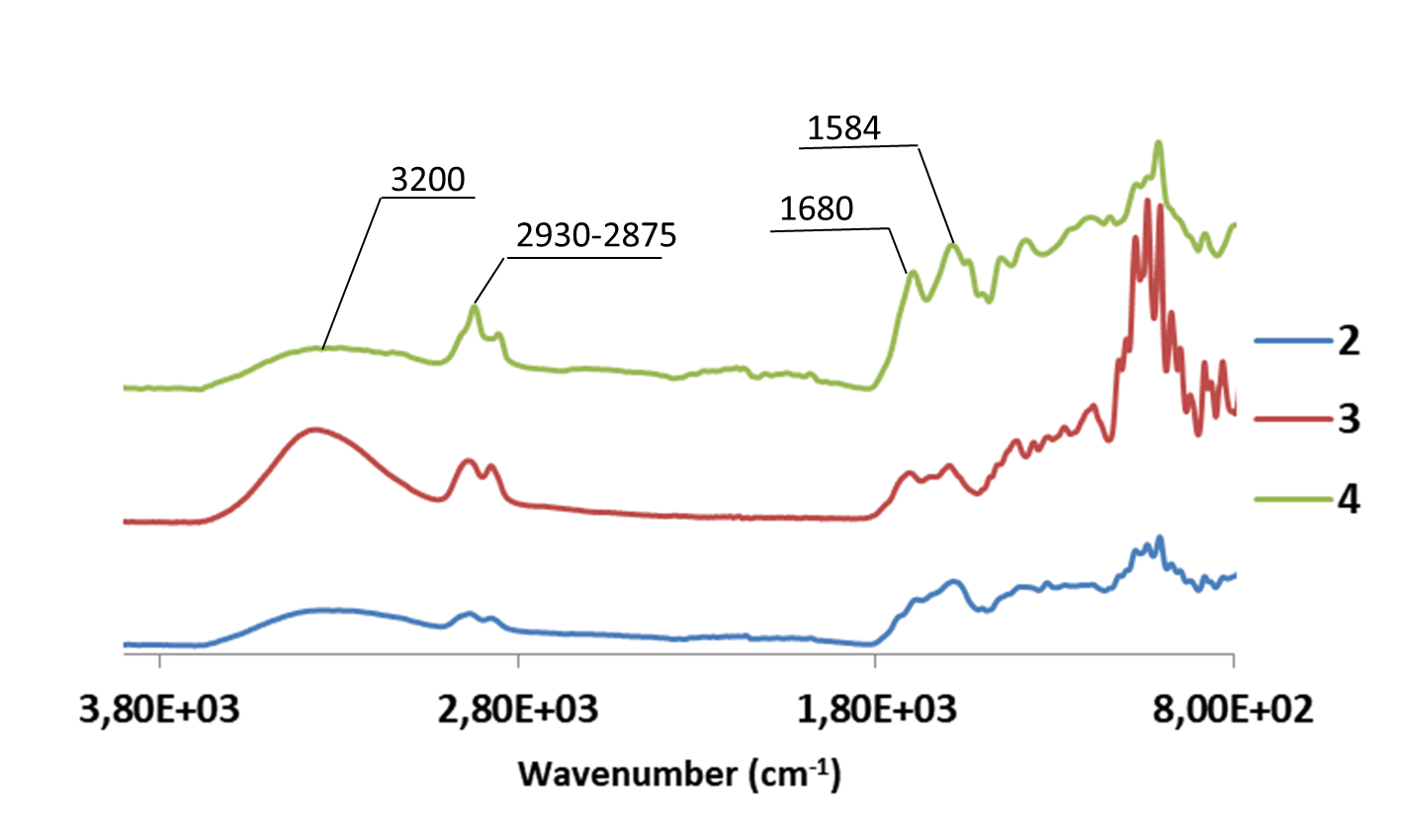


Fig S. 12: FTIR spectra of ROP products presented in Table 8, Entries 2-4

# Analysis of the PHUs

This section is relative to PHUs characterizations: FTIR (Fig S. 13), SEC (Fig S. 14-15), ^1^H- NMR (Fig S. 16-19), ^13^C- NMR (Fig S. 20-23), TGA (Fig S. 24) and DSC (Fig S. 25).

## FTIR analyses

FTIR analysis of the PHUs shows the absence of the C=O stretching peak at 1780 cm^-1^ from cyclic carbonate. The results of this analysis show the full conversion of the initial monomer. SEC analysis presents a large peak for all PHUs. It is a direct consequence of the different side reactions, which lead to a large Ð.**
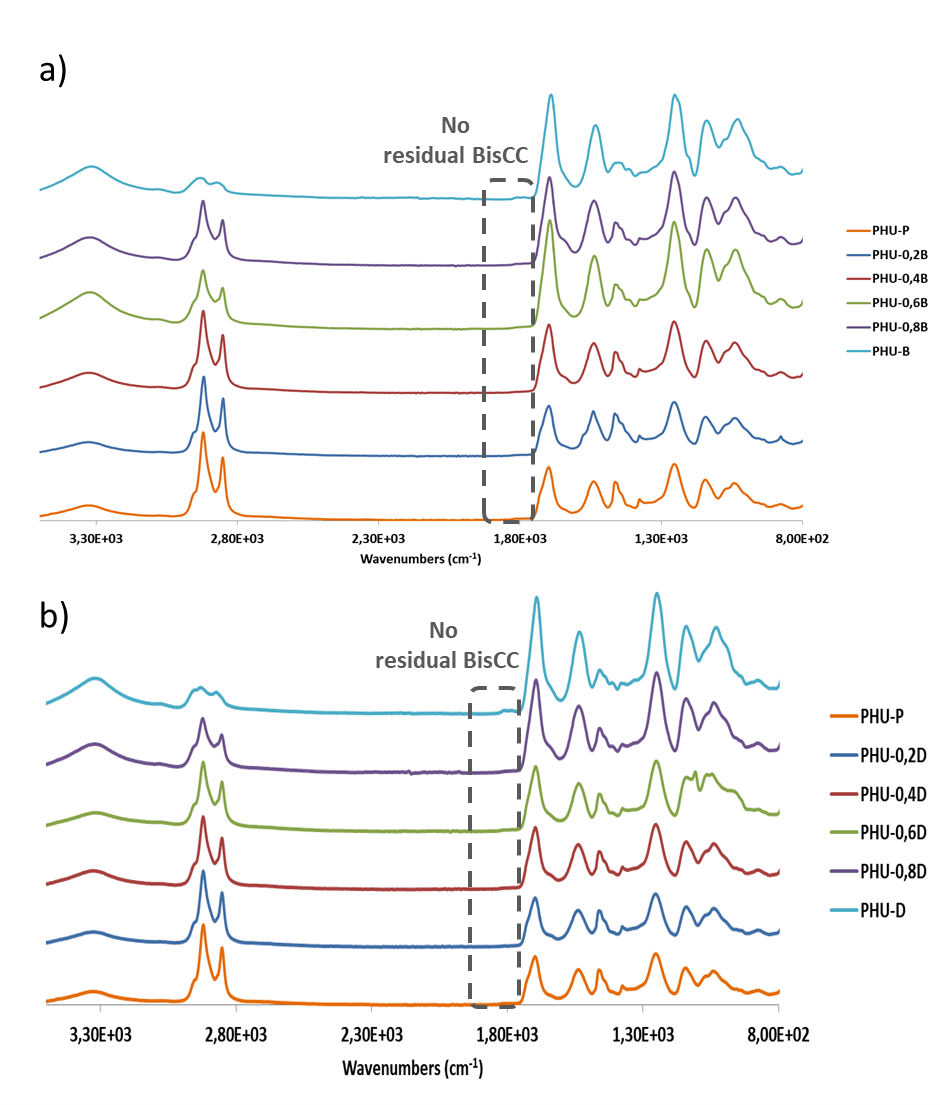
**

Fig S. 13: FTIR analysis of PHUs

## SEC analyses


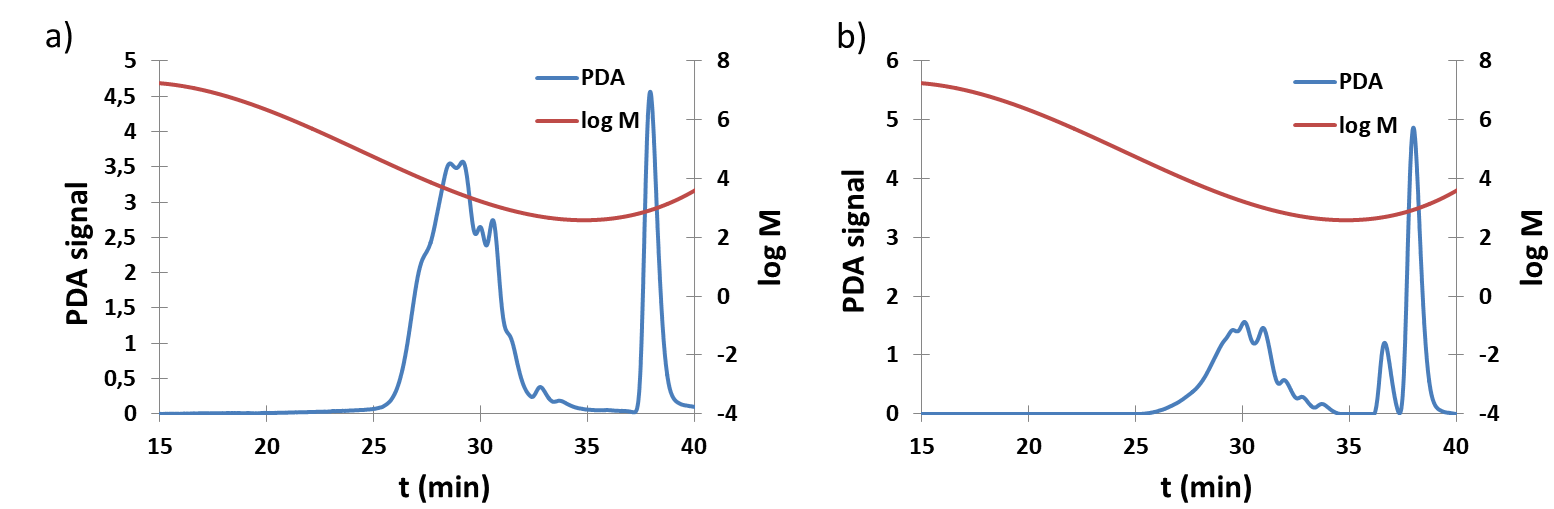


Fig SI 14: SEC curves a) PHU-P and b) PHU-H


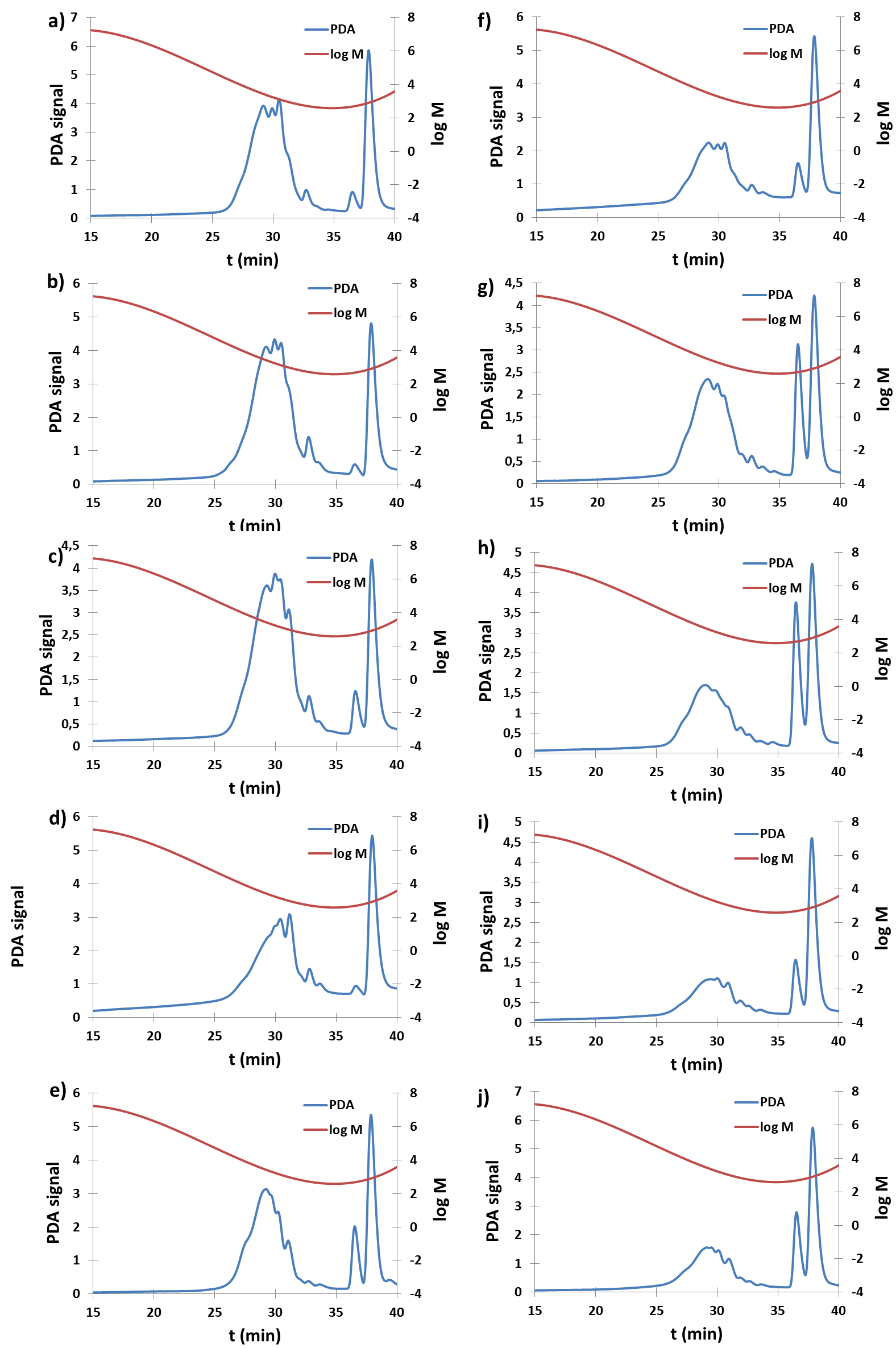


Fig S. 15: SEC curves of a) PHU-0.2B, b) PHU-0.4B, c) PHU-0.6B, d) PHU-0.8B, e) PHU-B, f) PHU-0.2D, g) PHU-0.4D, h) PHU-0.6D, i) PHU-0.8D, j) PHU-D

## ^1^H-NMR spectra

**
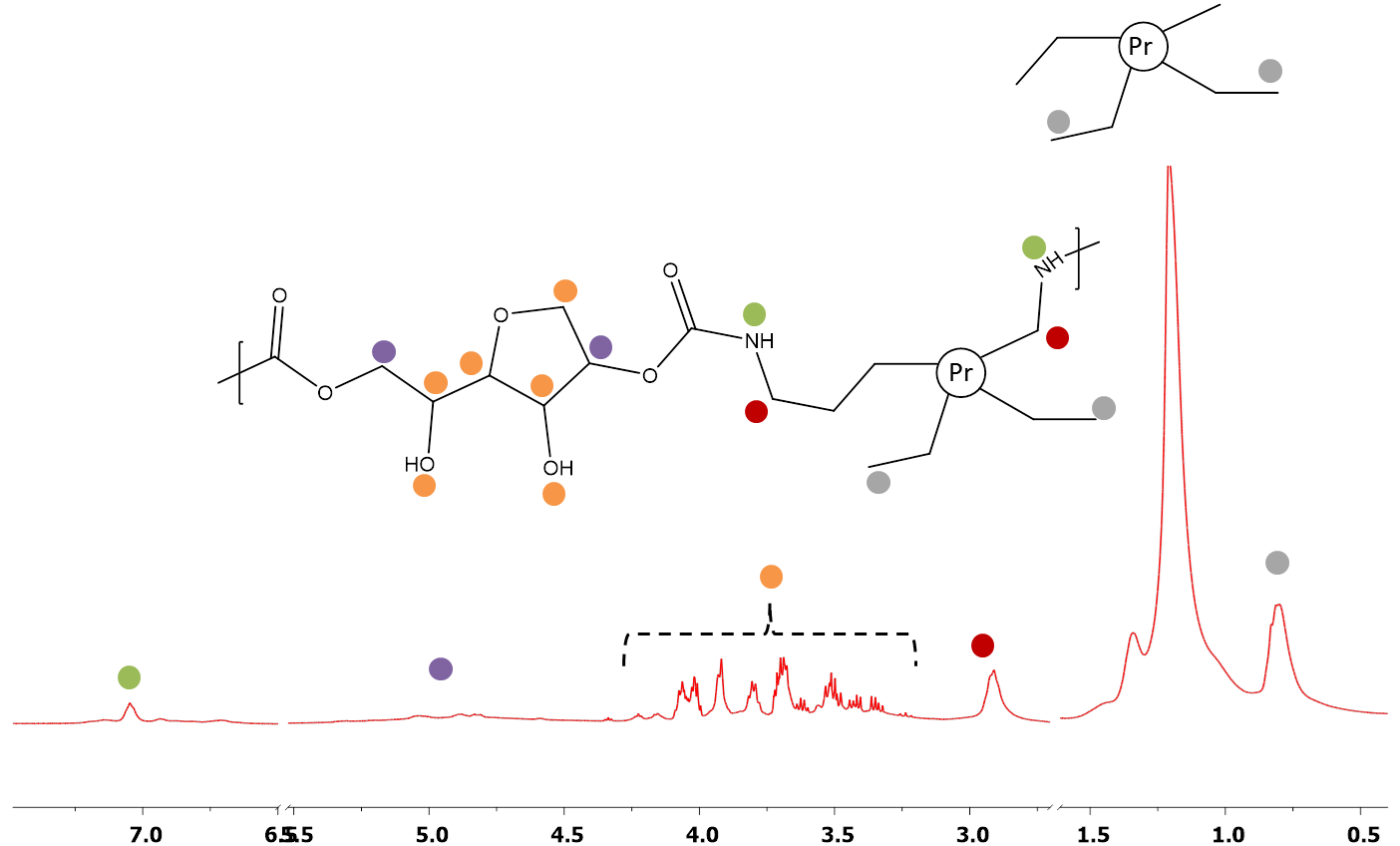
**

Fig S 16:^1^H-NMR of PHU-P sample

**
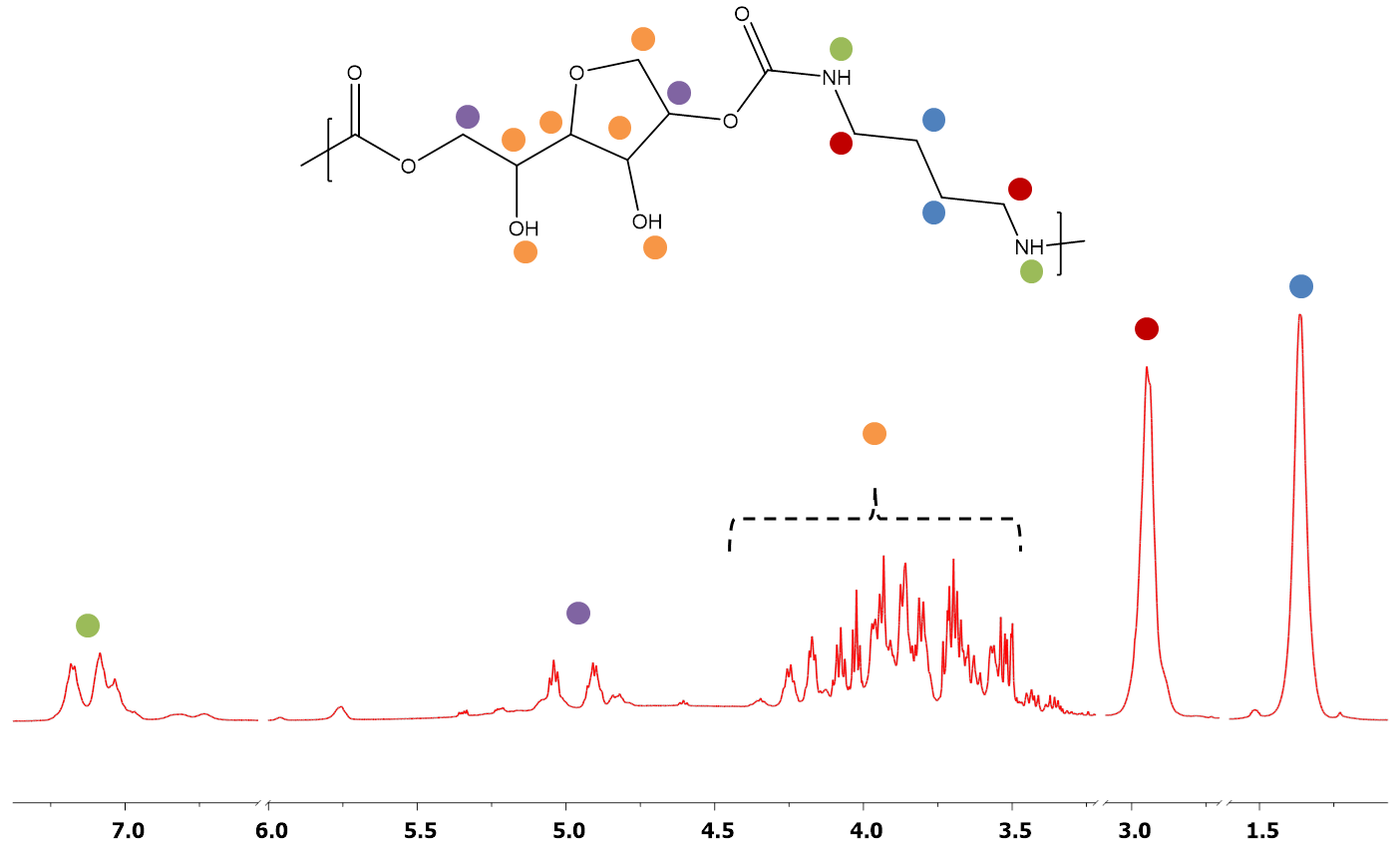
**

Fig S. 17: ^1^H-NMR of PHU-B sample

**
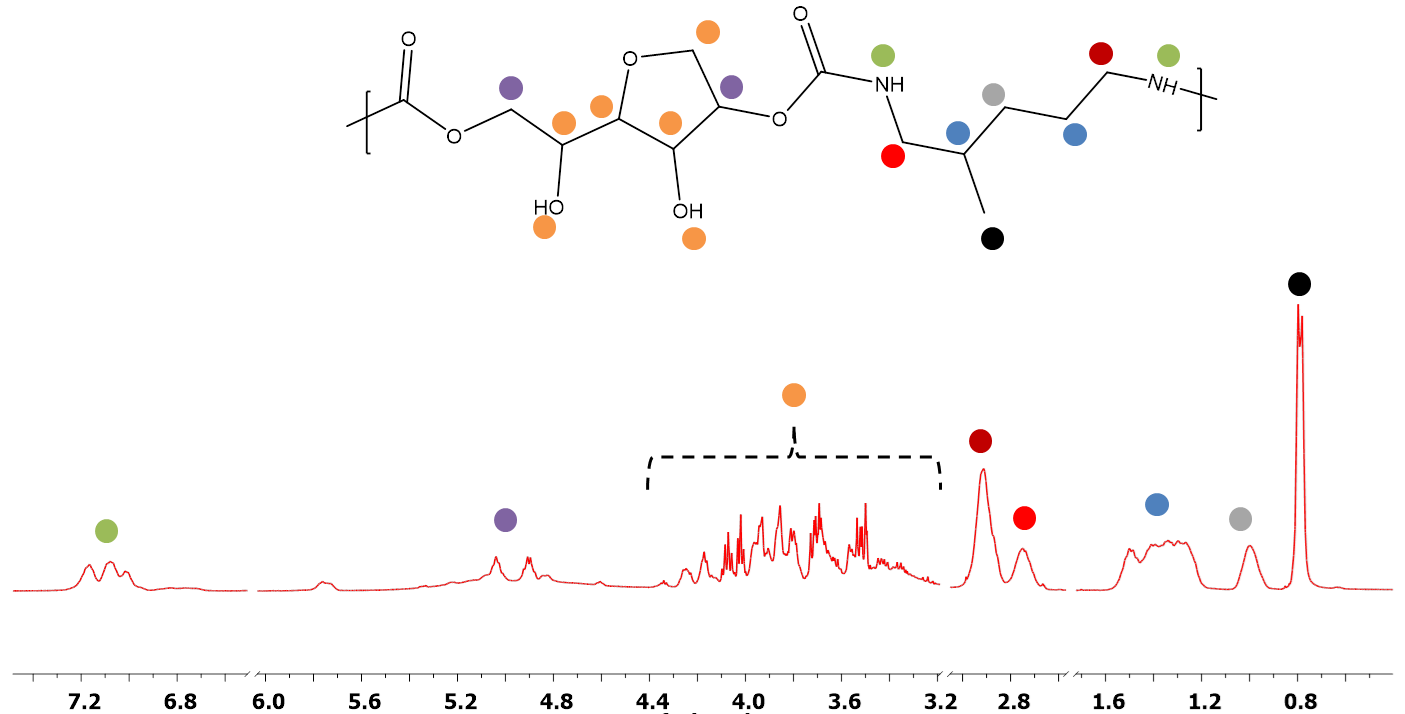
**

Fig S. 18: ^1^H-NMR of PHU-D sample

**
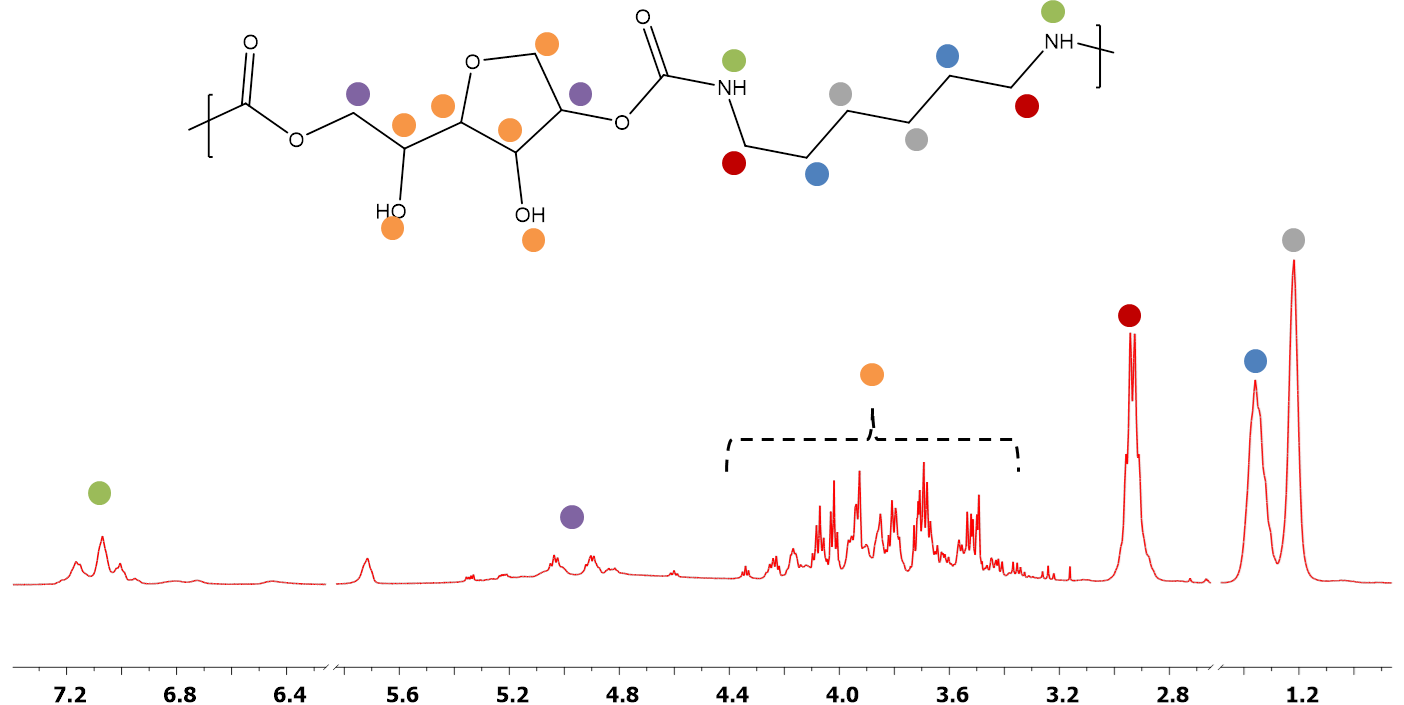
**

Fig S. 19: ^1^H-NMR of PHU-H sample

## ^13^C-NMR spectra

**
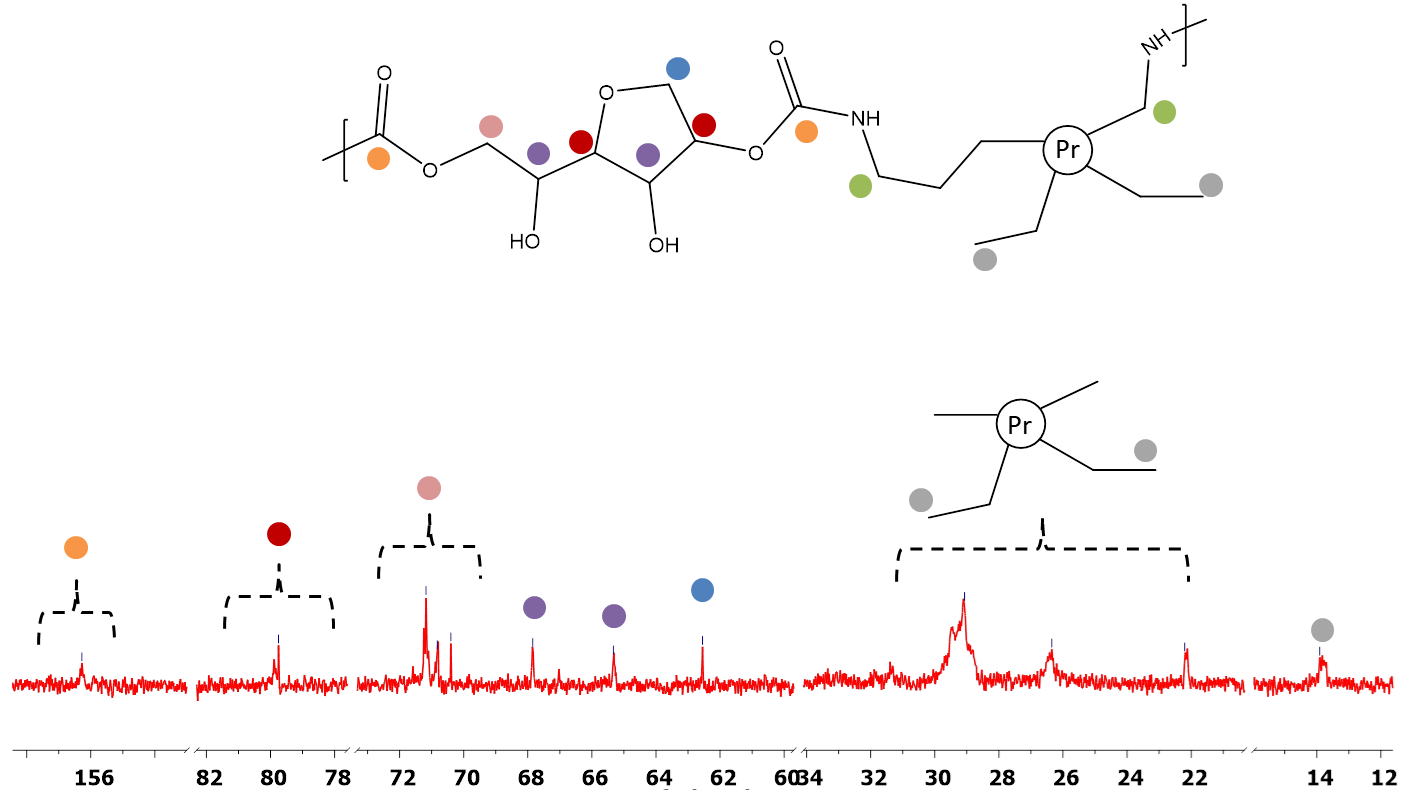
**

Fig S. 20: ^13^C-NMR of PHU-P sample

**
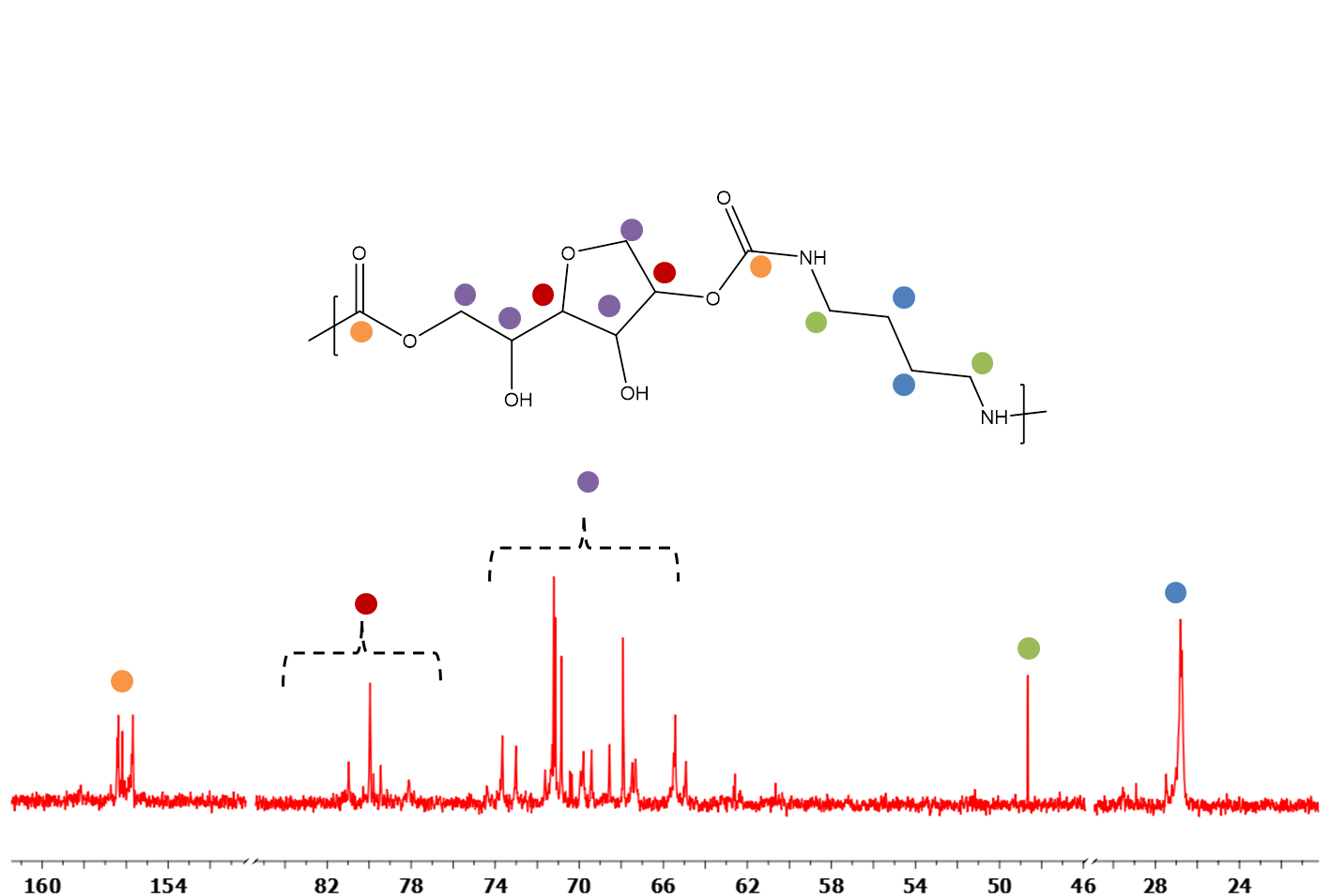
**

Fig S. 21: ^13^C-NMR of PHU-B sample

**
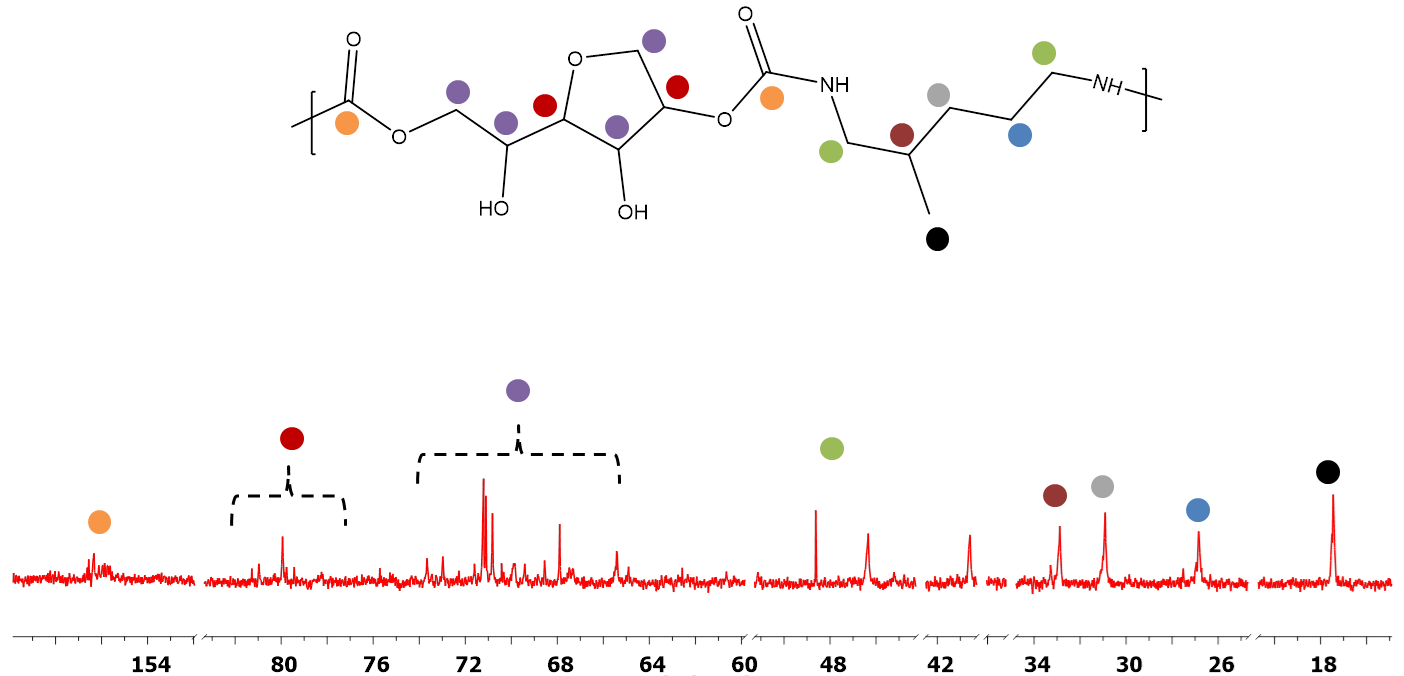
**

Fig S 22: ^13^C-NMR of PHU-D sample

**
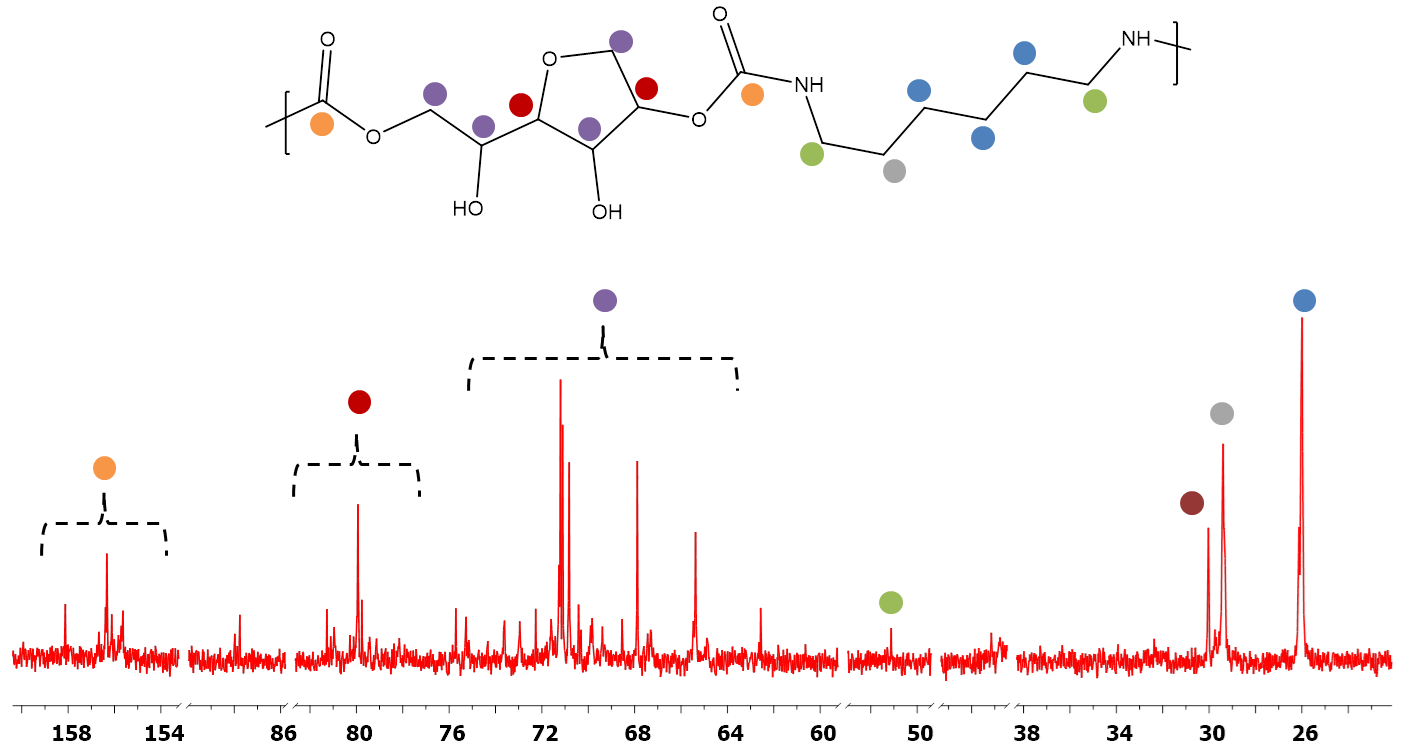
**

Fig S. 23: ^13^C-NMR of PHU-H sample

## ATG and DSC analysis of PHUs samples


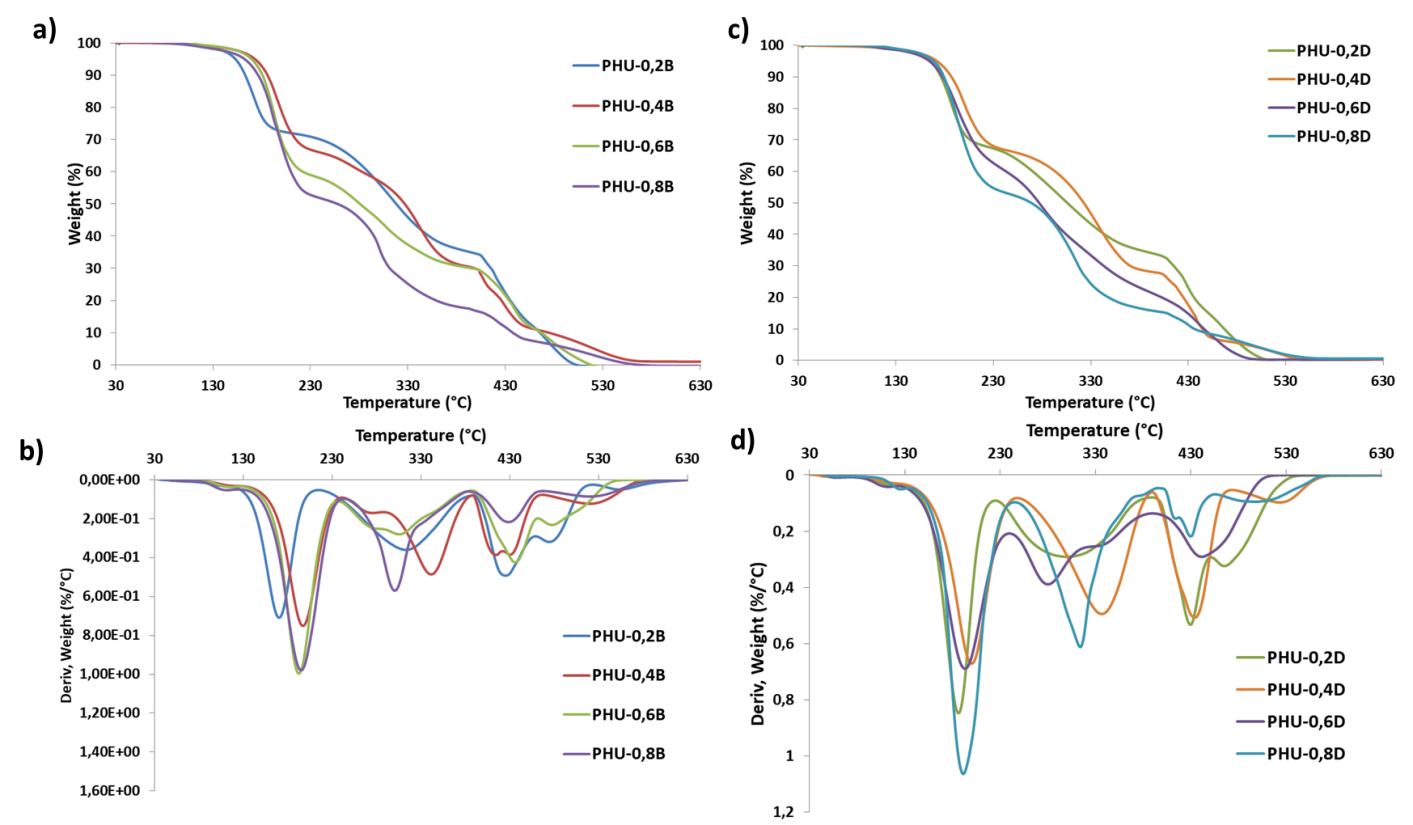


Fig S. 24: a) TGA and b) DTG curves of PHU-0.2B, PHU-0.4B, PHU-0.6B, PHU-0.8B samples; b) TGA and c) DTG curves of PHU-0.2D, PHU-0.4D, PHU-0.6D, PHU-0.8D samples


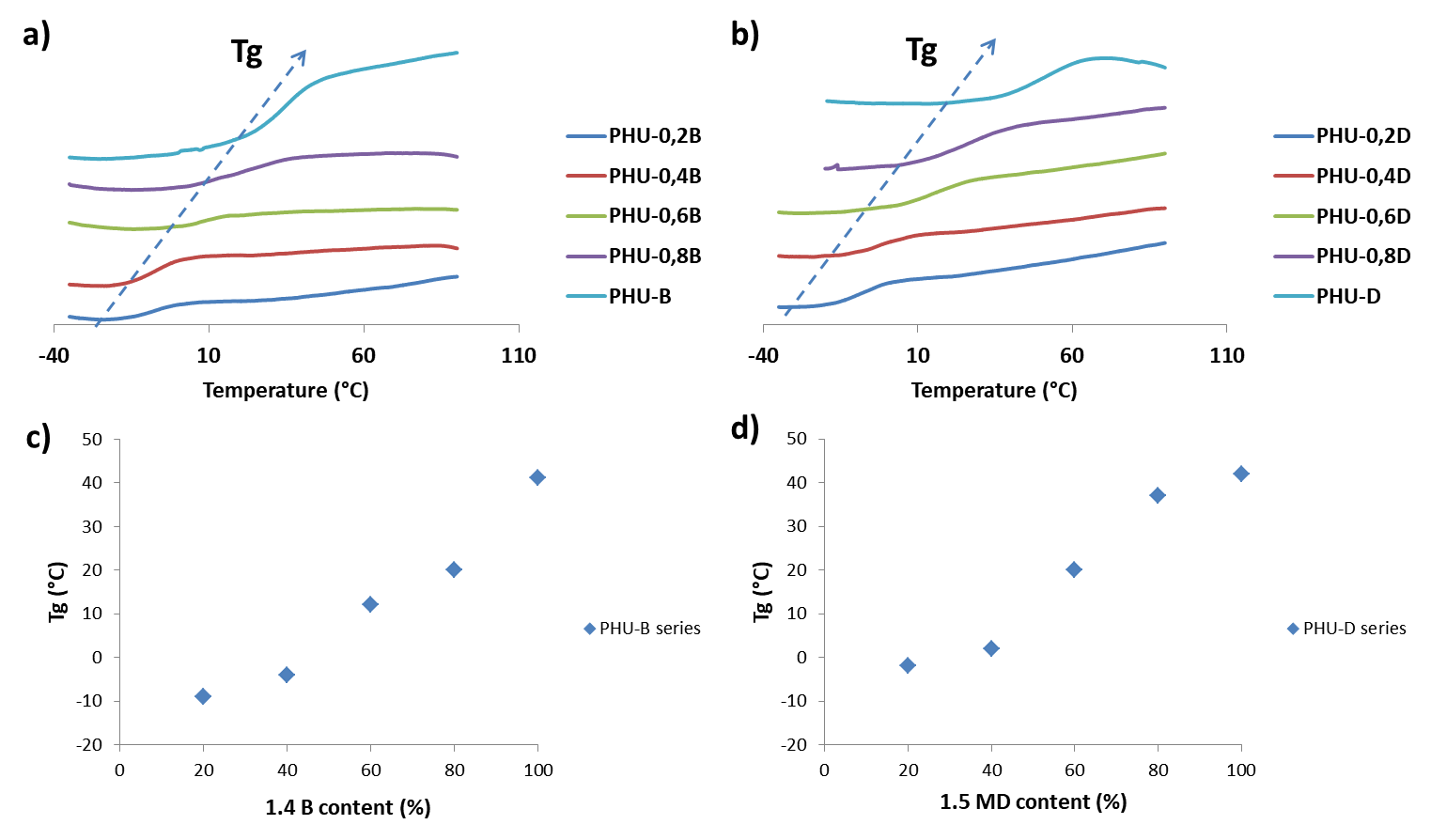


Fig S. 25: a) DSC curves of PHU-0.2B, PHU-0.4B, PHU-0.6B, PHU-0.8B, PHU-B samples and b) PHU-0.2D, PHU-0.4D, PHU-0.6D, PHU-0.8D, PHU-D samples, c) Tg evolution in function of the 1.4 B content compared to predicted Tg obtained by Fox Law, d) Tg evolution in function of the 1.5 MD content compared to predicted Tg obtained by Fox Law

DSC curves of the PHUs obtained from DDA and 1.4 B (Fig S.25, a) or 1.5 MD (Fig S.25, b) present a conventional increased of the Tg when the DDA content decrease. The Tg were plotted in function of the short diamine content (%) in Fig S.25, c-d. The theoretical Tg were determined by the Fow Law (Equation S.1)^(1)^.

$$\frac{1}{Tg}=\frac{W_{a}}{{Tg}_{a}}+\frac{W_{b}}{{Tg}_{b}} (S.1)$$

Where W_a_ and W_b_ are the weight fractions of components a and b, Tg_a_ and Tg_b_ are the glass transition of homopolymers a and b respectively in Kelvin, respectively. Tg is the predicted Tg of the corresponding copolymer obtained in Kelvin.

The Fox law is particularly relevant for periodic copolymer even if it is well known that the Fox law generally overestimates copolymers Tg’s. It is thus normal to observe a variation between the predicted Tg and the experimental data. By fitting the experimental data with a linear fit, it was determined that the Tg’s of PHU obtained from 1.4 B and 1.5 MD, respectively, evolved according to a first order polynomial law (Equation S. 2-3).

$$Tg=51.5x-21 R^{2}=0.96 (S.2)$$

$$Tg=87.5x-33 R^{2}=0.99 (S.3)$$

Where x is the short diamine content in %wt.

**ESI References:**

(1) Fox T.G., Influence of diluent and of copolymer composition on the glass temperature of a polymer system, Bull Am Phys Soc, **1956**, 2, 123
